# Identification of Climate-Smart Bread Wheat Germplasm Lines with Enhanced Adaptation to Global Warming

**DOI:** 10.3390/plants12152851

**Published:** 2023-08-02

**Authors:** Anil Patidar, Mahesh C. Yadav, Jyoti Kumari, Shailesh Tiwari, Gautam Chawla, Vijay Paul

**Affiliations:** 1 Division of Genomic Resources, Indian Council of Agricultural Research (ICAR)-National Bureau of Plant Genetic Resources, New Delhi 110012, India; anil.patidar@icar.gov.in (A.P.); shailu_2311@yahoo.co.in (S.T.); 2 Post-Graduate School, ICAR-Indian Agricultural Research Institute, New Delhi 110012, India; 3 Division of Germplasm Evaluation, ICAR-National Bureau of Plant Genetic Resources, New Delhi 110012, India; jyoti.kumari@icar.gov.in; 4 Division of Nematology, ICAR-Indian Agricultural Research Institute, New Delhi 110012, India; gchawla@iari.res.in; 5 Division of Plant Physiology, ICAR-Indian Agricultural Research Institute, New Delhi 110012, India

**Keywords:** bread wheat, climate-smart germplasm, adaptation, global warming, heat tolerance

## Abstract

Bread wheat (*Triticum aestivum* L.) is widely grown in sub-tropical and tropical areas and, as such, it is exposed to heatstress especially during the grain filling period (GFP). Global warming has further affected its production and productivity in these heat-stressed environments. We examined the effects of heatstress on 18 morpho-physiological and yield-related traits in 96 bread wheat accessions. Heat stress decreased crop growth and GFP, and consequently reduced morphological and yield-related traits in the delayed sown crop. A low heat susceptibility index and high yield stability were used for selecting tolerant accessions. Under heatstress, the days to 50% anthesis, flag-leaf area, chlorophyll content, normalized difference vegetation index (NDVI), thousand grain weight (TGW), harvest index and grain yield were significantly reduced both in tolerant and susceptible accessions. The reduction was severe in susceptible accessions (48.2% grain yield reduction in IC277741). The plant height, peduncle length and spike length showeda significant reduction in susceptible accessions, but a non-significant reduction in the tolerant accessions under the heatstress. The physiological traits like the canopy temperature depression (CTD), plant waxiness and leaf rolling were increased in tolerant accessions under heatstress. Scanning electron microscopy of matured wheat grains revealed ultrastructural changes in endosperm and aleurone cells due to heat stress. The reduction in size and density of large starch granules is the major cause of the yield and TGW decrease in the heat-stress-susceptible accessions. The most stable and high-yielding accessions, namely, IC566223, IC128454, IC335792, EC576707, IC535176, IC529207, IC446713 and IC416019 were identified as the climate-smart germplasm lines. We selected germplasm lines possessing desirable traits as potential parents for the development of bi-parent and multi-parent mapping populations.

## 1. Introduction

Bread wheat (*Triticum aestivum* L., 2n = 6X = 42, BBAADD), an important cereal crop, is a staple food for 40% of the world’s population [1]. It provides 20% of the total dietary calories consumed globally [2], and contributes proteins, vitamins and dietary fiber to the human diet and phytochemicals for human health benefits [3]. Wheat was cultivated on 219.0 million ha land with a global production of 760.9 million tons during 2020 and has contributed 8% to the world’s food basket [4].The demand of wheat is expected to rise by 60% from today’s level by 2050 and its production is expected to decrease by ~30% during this period due to extreme weather events [5]. Climate changes have impacted agriculture production and productivity globally during the past decades and seriously threatened the food supply [6,7]. Therefore, major advances in global food systems are required to ensure food security to the burgeoning human population, which is expected to reach 10 billion by 2050 and for sustainable development [8]. With increasing climate instability due to global warming, agricultural productivity will continue to be negatively impacted [9]. Studies suggest that every 1 °C rise in the average global surface temperature will lead to a decline in wheat yields from 4.1 to 6.4% worldwide and 8.0% in India [10].

The narrow genetic base of elite cultivars necessitates the screening of germplasm conserved in genebanks to enhance resilience against stressors [11]. The efficient utilization of germplasm resources is crucial for enhancing the genetic gains to address the challenges posed by global warming [12]. Genetic diversity is paramount for crop improvement, and for bread wheat, this resides in three genomes, which were constituted by the polyploidization of ancestral diploid species [13]. Bread wheat originated in Fertile Crescent after a few events of initial allopolyploidization and it spread to all continents except Antarctica [14]. During the course of evolution and, later on, its global cultivation, bread wheat has adapted to various agro-ecologies from temperate (cold) to sub-tropical (hot and dry) and tropical (hot and humid) environments. The widely adapted germplasm with stress tolerance conserved in genebanks of national and international research institutes (e.g., World Wheat Collection, CIMMYT, Mexico), needs to be utilized in breeding programs for further enhancing the genetic gains in wheat [15,16].

The Indo-Gangetic Plains (IGP) region of India contributes about 15% of global wheat production. However, about 51% of its area might be re-classified as a heat-stressed (HS) short-season production mega-environment by 2050 [17]. Globally, heatstress during the grain filling period (GFP) is a major yield-reducing factor [18]. Heatstress during GFP, commonly referred to as terminal-heat stress, adversely affects plant growth and grain yield. Late-sown wheat is invariably exposed to terminal-heat stress, resulting in significant yield losses [19,20]. The delayed sowing of wheat is common in the IGP region and hence it endures heatstress during GFP [21]. Every 1 °C rise in temperature above the optimum of 28 °C during GFP has resulted in yield losses of 3–17% in the Great Plains of USA and the Eastern IGP of India [22,23]. Hence, study on the impact of heatstress on the productivity of wheat in these regions has emerged as a top priority in the climate change scenario.

Several adaptive morpho-physiological traits like early ground cover, epicuticular wax, leaf rolling, stay-green, biomass and flag-leaf area contribute to heat-stress tolerance in wheat [24,25]. Adaptive physiological traits such as CTD, cell membrane thermo-stability, NDVI, chlorophyll content and fluorescenceare associated with heat-stress tolerance and significantly contribute to the performance of tolerant wheat lines in an HS environment [26,27]. Heatstress during crop growth and grain development significantly reduces morphological and yield-related traits, viz., plant height, tiller number, peduncle length, spike length, spikelets and grains per spike, TGW and yield in wheat [28,29]. Endosperm shrinkage in grains of heat-stressed plants is a major cause of yield and TGW reduction [30,31]. Heatstress causes damage to the cellular structure and affects various metabolic pathways, mainly those related to membrane thermostability, photosynthesis and starch synthesis [32,33,34]. Each genotype responds to a changed environment differently due to its genetic makeup and interaction with the environment [19,35]. The genotypes, which maintain a high TGW and yield under an HS environment, seem to possess a higher tolerance to a hot environment [36,37]. The exposure of wheat plants, at anthesis and during GFP, to a higher than the optimal temperature, affects the grain development and, as a result, reduces productivity [38,39]. The knowledge of the source–sink relationship during grain development is critical for the selection of germplasm that can produce a better yield and quality under global warming [40]. Thus, an understanding of the sink– source relationship under heatstress in wheat germplasm could be useful for selecting tolerant lines.

Genotype–Environment (G × E) interactions are important factors in the expression of quantitative traits such as yield and its component traits. A stability analysis has been used to identify varieties with a superior performance and yield stability under different environments [41]. Germplasm offers the best opportunity to develop varieties with a small G × E interaction. Climate-smart varieties tolerate negative effects of climate change better and produce a higher yield and better quality in stressful environments. Screening a large genebank collection for tolerance to stresses is a right approach to develop climate- smart varieties [11]. The morpho-physiological traits have been used to screen germplasm for heat-stress tolerance in numerous studies worldwide [18,42]. The testing of diverse germplasm under an HS environment would be useful to understand plant responses to heatstress and the identification of superior lines for the development of mapping populations. Thus, aims of this study were to understand the impacts of heatstress on morpho-physiological and grain traits, to identify climate-smart germplasm and to select desirable parents for the creation of bi-parent and multi-parent mapping populations.

## 2. Materials and Methods

### 2.1. Plant Materials

Plant materials consisted of a subset of 96 bread wheat accessions (79 indigenous and 17 exotic collections), selected from a large genebank collection of wheat [43], which included released varieties for the late-sown condition, trait-specific germplasm and genetic stocks. Seeds of wheat accessions were obtained from the working collection of the Indian National Gene Bank (INGB) at the ICAR-National Bureau of Plant Genetic Resources (NBPGR), New Delhi. Passport data of wheat accessions are provided in Supplementary Table S1. The geo-referencing of wheat accessions on the world map was performed using the software ‘DIVA-GIS’ [44]. The germplasm chosen for the present study were genetically diverse and represented all wheat growing zones of India (Supplementary Figure S1).

### 2.2. Experimental Site, Design and Weather Conditions

Field experiments were conducted at Experimental Farm, ICAR-NBPGR, New Delhi, situated at 28.649° N latitude, 77.152° E longitude and 220 m altitude, during the *Rabi* seasons of years 2018–2019 and 2019–2020. The farm area lies in the North–Western Plain Zone (NWPZ) of wheat production with a semi-arid and sub-tropical climate and sandy- loam alluvial soil, slightly alkaline in pH and low in organic matter content. In each crop season, two sowing dates were (i) normal (sown in first week of December; non-stressed (NS) environment) and (ii) late-sown wheat (sown in first week of January; HS environment). Thus, in late-sown wheat, accessions were exposed to heatstress during GFP. The combination of year and sowing dates made our field experiments have four environments, which included two NS and two HS environments for testing the stability of wheat germplasm under heatstress. The field trials were laid out in an Augmented Block Design (ABD) with fiveblocks, where four checks, namely, Raj3765, HD2932, WR544 and HD2967, were randomized and replicated in each block. Each experimental plot consisted of three rows of a 2.0 m length with 25 cm spacing between rows (1.5 m^2^ area). The standard crop management practices for irrigated ecology were followed for raising a healthy wheat crop. Weather parameters like the temperature and rainfall were recorded during the crop period of 2018–2019 and 2019–2020. Crop duration was expressed in standard meteorological weeks (SMW).

### 2.3. Field Phenotyping and Data Recording

The morpho-physiological and yield traits related to heat-stress tolerance were recorded as per the Manual on Physiological Breeding II: A field guide to wheat phenotyping [45]. The traits were recorded during different growth phases of wheat trials (Table 1).

### 2.4. Scanning Electron Microscopy

Mature grains from heat-stress tolerant and susceptible accessions of bread wheat were transverse sectioned into three small pieces. The middle sections were mounted on an aluminum stub using double-side adhesive carbon tape. The specimens were uniformly coated with a thin layer of gold–palladium using an Emitech SC7620 sputter coater. Specimens were examined under a Scanning Electron Microscope (SEM, Model Tescan Vega3, Tescan Analytics, Fuveau, Alpes-Côte d’Azur, France) operated at 10.0 kV using a secondary electron detector. The aleurone layer, endosperm cells and starch granules were observed, and their images were captured.

### 2.5. Statistical Analysis

Adjusted mean values were used for the statistical analyses of ABD trial data of 18 traits recorded under NS and HS environments over 2 years. An analysis of variance (ANOVA) was performed following a test of the homogeneity of variances and applying Aitkin’s transformation using SAS software version 9.4 [46]. Pearson’s correlation coefficients (r) were derived using IBM SPSS statistics software version 20.0 [47] for both NS and HS environments. A cluster analysis was performed with Ward’s minimum variance using Euclidean distance matrices. The dendrogram was constructed using the unweighted paired group method of the arithmetic averages (UPGMA) algorithm. We categorized the accessions as tolerant or susceptible based on the heat susceptibility index (HSI). HSI was computed based on grain yield (GY) data using the formula [48] HSI = (1 − X_a_/X_b_)/(1 − Y_a_/Y_b_), where X_a_ and X_b_ are mean values of the GY of an individual accession under the HS and the NS environment, respectively; Y_a_ and Y_b_ are mean values of the GY of all accessions under the HS and the NS environment, respectively.

We used HSI values to categorize wheat accessions as highly tolerant (HSI < 0.50), tolerant (HSI = 0.51–1.0), susceptible (HSI = 1.0–1.50) and highly susceptible (HSI > 1.50). The phenotypic variance (σ^2^_ph_), genotypic variance (σ^2^_g_), phenotypic coefficient of variation (PCV), genotypic coefficient of variation (GCV), broad sense heritability (*H*^2^) and genetic advance (GA) were calculated on mean data of four checks using SAS software.

Heritability (*H^2^*) % = (σ^2^_g_/σ^2^_ph_) × 100 and genetic advance (GA) = H × k × σ_ph_, where σ_ph_ is the phenotypic standard deviation and k is the constant 2.06 at 5% selection intensity, were computed. The diversity estimates were derived using the Shannon–Weiner index [49].

$$\mathrm{Shannon}-\mathrm{Weiner}\;\mathrm{index}(H\prime )\;=-\sum_{i=1}^{n}\mathrm{pi}\;\ln\left(\mathrm{pi}\right)$$

where n is the number of phenotypic classes for a character, P_i_ is the relative frequency in the ith class of the jth trait and ln is the natural logarithm of P_i_. The extent of diversity was interpreted as H′ < 0.5: low, H′ = 0.5 to 1.0: high and H′ > 1.0: very high. The traits showing H′ > 1.5 revealed a great genetic diversity among the accessions.

We performed a stability analysis usingthe Eberhart and Russell (1966) model [50] with Windostat software (IndoStat Services, Hyderabad, India). The stability model used is Y_ij_ = µ_i_ + β_i_I_j_ + δ_ij_, where Y_ij_ is the mean of the ith genotype in the jth environment, µ_i_ is the ith genotype mean over all environments, β_i_ is the regression coefficient of the ith genotype, I_j_ is the environmental index and δ_ij_ is the deviation from the regression of the ith genotype at the jth environment. The stable genotypes have a higher mean than the population mean, regression coefficient (β_i_ = 1) and small S^2^d_i_. These genotypes are well adapted to both the environments. The genotypes having a higher mean, β_i_ > 1 and a small S^2^d_i_ are adapted to the favorable (NS) environment. The genotypes with a higher mean, β_i_ < 1 and a small S^2^d_i_ are specially adapted to the unfavorable (HS) environment. Four categories of accessions were made, viz., highly tolerant, tolerant, susceptible and highly susceptible based on the mean, stability and HSI.

## 3. Results

### 3.1. Weather Conditions during Crop Seasons

**Figure 1:**
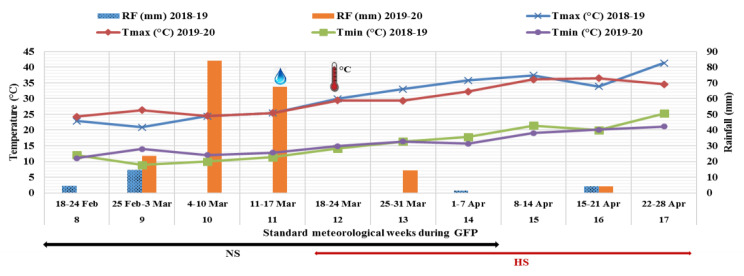
Temperature and rainfall variation during grain filling period (GFP; from anthesis to physiological maturity) in both non–stressed (NS) and heat-stressed (HS) environments in wheat crop seasons of 2018–2019 and 2019–2020. RF–rainfall, Tmax—maximum temperature, Tmin—minimum temperature.

The maximum and minimum temperatures and rainfall during the GFP of 2018–2019 and 2019–2020 crop growing seasons are presented in Figure 1. GFP started from 8 SMW (18–24 February) and completed during 14 SMW (1–7 April) in the normal sown (NS) crop, while it was from 12 SMW (18–24 March) to 17 SMW (22–28 April) for the late-sown (HS) crops. Light to moderate rainfall occurred during GFP under the NS environment in 2018–2019, but it was moderate to heavy rainfall during 2019–2020. The light rainfall during GFP slightly lowered the rising day and night temperatures under the HS environment during both the crop seasons. The wheat crops of the HS environment (sown in January) were exposed to mean day temperatures of above 35 °C in 2018–2019 and 33 °C in 2019–2020 during the grain development. The night temperature was also higher during GFP under the HS environment than the NS environment during both the crop seasons. On average, the maximum temperature was 7.7 °C higher in 2018–2019 and 5.7 °C in 2019–2020 under the HS environment. Thus, the air temperature during GFP for the late-sown crop was significantly higher than the optimum temperature needed for grain development (22–25 °C). Hence, the late-sown crops were exposed to severe heatstress during GFP.

### 3.2. Crop Growth and Genetic Variability

The differences in the climatic conditions in the environments were reflected by the variations observed for the phenological and agronomic traits, as presented in Table 2. Significant differences were observed for DA, PH, PL, FLA, SL, NSS and GFP between environments. Bread wheat accessions showed significant differences for all the traits except CTD, under both NS and HS environments (Supplementary Table S2). CTD showed a large variation due to the year effect and hence exhibited a non-significant variation for the genotype. The other physiological traits like NDVI and MSI also showed a greater variation due to the year than the genotype. Thus, the variation due to the year was significant for almost all the traits except CC, LR, NSS, HI and YPP, under both NS and HS environments. Then genotype × year (G × Y) interaction showed non-significant differences in most of the traits. NDVI had a significant G × Y interaction in both NS and HS environments, while TGW showed a significant G × Y interaction in the NS environment. Morphological traits, namely, PH, PL, SL and GL, showed a significant G × Y interaction under heatstress. Thus, the morpho-physiological and yield-related traits showed a wide-range of phenotypic variability under both NS and HS environments. Check variety WR544 (IC296383) had the earliest anthesis on 83.4 and 66.6 days in normal and late-sown trials, respectively.

Accessions IC393878 and IC252725 showed the highest GFP of 39.5 and 32.0 days under NS and HS environments, respectively. Accession EC577013 had the tallest plants (150.9 cm) under NS, while accession EC576585 had the tallest plants (127.0 cm) under the HS environment. However, accession IC335792 produced the shortest plant (84.9 cm) under NS and accession IC443653 had the shortest plants (73.2 cm) under the HS environment. Accession IC277741 produced the highest grain yield (802.5 g) in the NS environment, while accession IC566223 produced the highest grain yield (598.1 g) under the HS environment. The lowest grain yield (300.1 g) was produced in accession IC542509 under NS, whereas accession IC539287 produced the lowest yield (176.7 g) under the HS environment. Accession IC573461 showed the highest TGW (52.2 g) under the NS environment, while accession IC539221 produced the highest TGW (46.6 g) under the HS environment. Accession IC443653 showed the highest harvest index (48.0%) under the HS environment.

**Figure 2:**
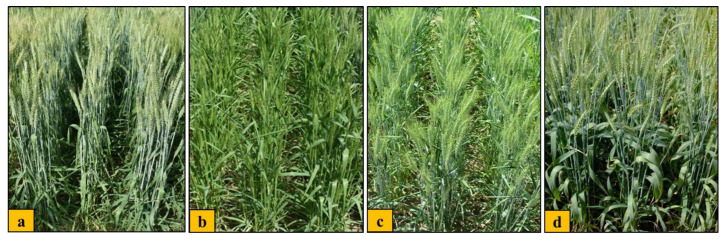
Adaptation to terminal heatstress by highly tolerant accessions of bread wheat germplasm during grain filling period.Comparisons ofaccession IC529207 (highly tolerant) with highest plant waxiness (score 10) (**a**) vs. accession IC252431 (highly susceptible) showing the least plant waxiness (score 1) (**b**), and accession IC416019 (highly tolerant) showing the highest leaf rolling (score 10) (**c**) vs. IC553599 (highly susceptible) showing the least leaf rolling (score 4) (**d**).

Bread wheat accessions exhibited a significant variation for most of the physiological traits. They showed a high variability in physiological traits such as plant waxiness and leaf rolling (Figure 2). Accessions IC528965 and IC529207 showed the highest waxiness (score 10) under both the environments. Accession IC416019 showed the highest leaf rolling (score 9.5) under NS, whereas accessions IC416019 and IC416055 showed the highest leaf rolling (score 10) under the HS environment. Accession IC573461 showed the highest CC (38.5) under NS, while accession IC252444 had the highest CC (37.2) in the HS condition. However, accession IC542547 possessed the lowest CC under both the environments. Accessions IC542509 and IC566223 showed the highest NDVI under both NS (0.72) and HS (0.64) environments. CTD was higher under HS than the NS environment. Accession EC190962 showed the highest value of CTD (9.7 °C) under the NS environment, while accession IC542509 exhibited the highest CTD (12.0 °C) under the HS environment. The accessions IC277741 and 83 EC573527 exhibited the highest value for MSI (77.5%) under NS, while accession IC542544 showed the highest MSI (72.6%) under the HS environment. Detailed data for all the traits are shown in Supplementary Tables S3 and S4.

A wide range of genetic variability was recorded for most of the traits (Table 2). PCV was higher than GCV for all the quantitative traits. The highest PCV was observed for CTD (26.1%) under the HS environment followed by PW (23.8%) under the NS environment. The highest GCV was recorded for PW (19.6%) followed by LR (17.5%) under the NS environment. The estimates of a high *H*^2^ (>70%) were recorded for DA (84.9% under NS and 85.4% under HS) and GL (77.9% under NS and 89.3% under HS) in both environments; CC (75.9%) and PL (79.4%) in HS; and LR (71.4%) in the NS environment. A genetic advance was the highest for PW (33.2%) followed by LR (30.5%) under the NS environment.

The Shannon–Weiner diversity index (H′) revealed a high morpho-physiological diversity among studied bread wheat accessions considering all traits under NS (H′ = 1.56) and HS environments (H′ = 1.47). The most diverse traits were PW (H′ = 1.90), CTD (H′ = 1.88), NSS (H′ = 1.79) and NDVI (H′ = 1.78) under NS, while under the HS environment, NSS (H′ = 1.78), PW (H′ = 1.77), MSI (H′ = 1.70) and CTD (H′ = 1.69) displayed a high phenotypic diversity. The grain length showed the least variability under both NS (H′ = 1.19) and HS (H′ = 1.14) environments. The frequency distribution for 12 important phenological, physiological and yield-related traits, namely, DA, GFP, CC, CTD, NDVI, MSI, PW, LR, PH, PL, TGW and GY, also supported the existence of a widerange of genetic variability in the germplasm (Supplementary Figure S2).

### 3.3. Genetic Relationship in Wheat Accessions

**Figure 3:**
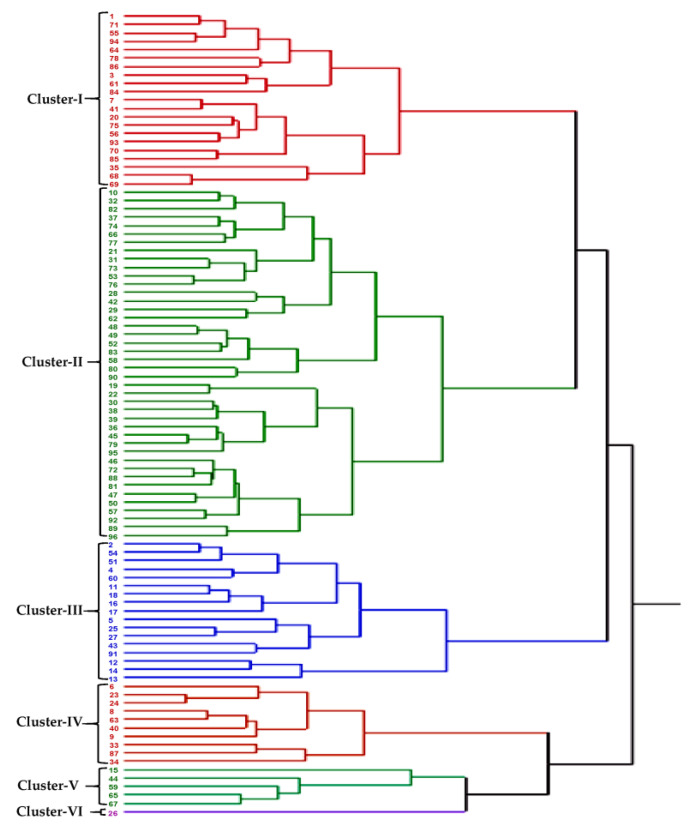
UPGMA dendrogram constructed with Ward’s minimum variance method for 96 bread wheat accessions using data of 18 morpho-physiological and yield-related traits recorded under heat-stress environment. Six clusters are marked on the left side of the dendrogram.

A UPGMA dendrogram grouped all the 96 wheat accessions into six major clusters using data of 18 morpho-physiological traits recorded under the HS environment (Figure 3).

Most of the accessions were included in the first three clusters, and cluster I, II and III consisted of 21, 42 and 17 accessions, respectively. The remaining three clusters grouped 16 accessions with cluster IV, V and VI having 10, 5 and 1 accessions, respectively. Most of the accessions adapted to heatstress, *viz*., accession IC566223, IC529207, IC535176, EC534487, IC252348, IC401976, IC075240, IC539531 and EC574731, grouped in cluster III along with two national check varieties, HD2932 and HD2967. Accession IC542509 took the longest time to 50% anthesis (119 days in NS and 94 days in HS environment), produced the lowest yield in both the NS and HS environments and formed a solitary accession in cluster VI. Accessions EC576585, IC536162, IC252999, EC190899, IC252867, IC536050, IC416075, IC572925, IC443653 and WR544 flowered early (<69 days) in the HS environment and were grouped in cluster I.

### 3.4. Correlations with Grain Yield

**Figure 4:**
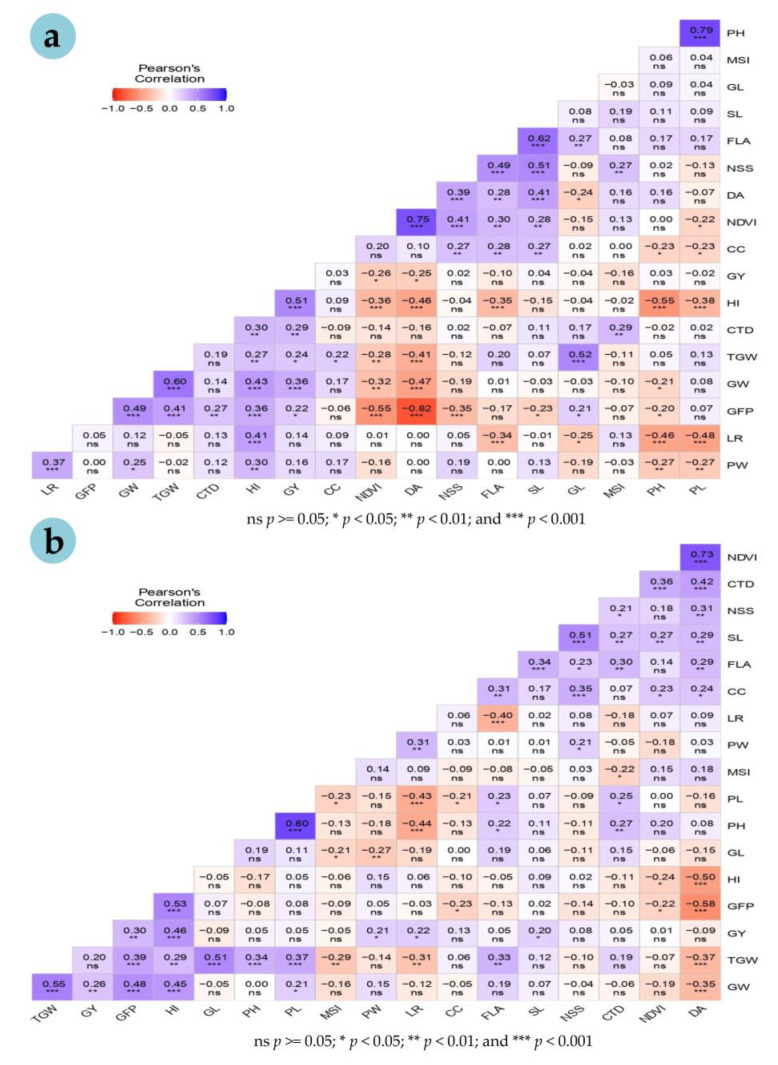
Pearson’s correlations coefficients (r) derived between 18 morpho-physiological and yield traits under non-stressed (**a**) and heat-stressed (**b**) environments in 96 bread wheat accessions.

Grain yield associated positively with GFP, PW, LR, SL, HI and GW under the HS environment (Figure 4a). However, under the NS environment, GY associated positively with CTD, GFP, HI, GW and TGW, but negatively with NDVI and DA (Figure 4b).

TGW showed positive associations with GFP, HI, GL and GW under both NS and HS environments. However, under heatstress, TGW was associated positively with PH, PL and FLA, but negatively with MSI, LR and DA. Strong positive correlations were recorded between PH and PL, and SL and NSS, under both the environments. GFP associated positively with GW, TGW and HI, but negatively with DA under both the environments. Similarly, DA also associated negatively with HI, TGW and GW under both environments. Among the physiological traits, positive associations were observed between PW and LR under both the environments. NDVI associated positively with both CC and CTD under the HS environment. The cooler canopy (CTD) showed positive correlations with DA, PH, PL, FLA, SL and NSS under the HS environment. However, CTD associated positively with MSI under NS and negatively under the HS environment.

### 3.5. Impact of HeatStress on Yield and Morpho-Physiological Traits

**Figure 5:**
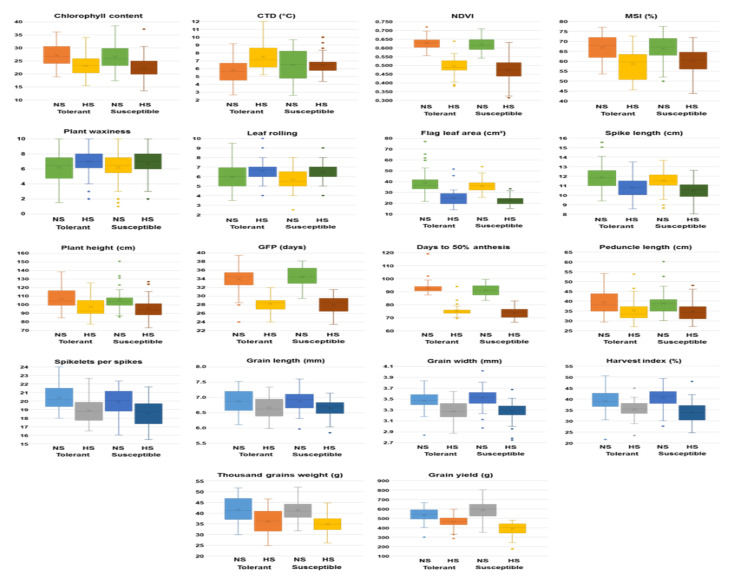
Boxplot distribution of variability assessed for 18 morpho-physiological and yield-related traits under non-stressed (NS) and heat-stressed (HS) environments in 96 bread wheat accessions categorized based on HSI values as tolerant (HSI < 1.0) and susceptible (HSI > 1.0).

Late sowing reduced the optimal period for crop growth and grain development. The unfavorable weather conditions prevailing under the HS environment had an adverse effect on the grain yield and its contributing traits, which showed a reduced expression. However, physiological adaptive traits, namely, CTD, PW and LR, showed an increased expression under the HS environment. Phenological traits like DA were reduced by 17 days. PH, PL and GFP were decreased by 9.7 cm, 4.1 cm and 6.0 days, respectively, under the heat-stress condition. Likewise, heatstress reduced FLA by 13.6 cm^2^. The heatstress prevailed, at the onset of the reproductive phase and during the grain development stage, decreased SL, NSS, GL and GW. The heatstress during GFP had an adverse effect on grain yield and TGW, and reduced these traits by 138.6 g and 6.0 g, respectively. Among the physiological traits, CC, NDVI and MSI were reduced by 4.2, 0.14 and 7.2%, respectively, under heatstress. However, CTD increased from 6.2 to 6.9 °C under the HS environment to maintain an optimal sub-cellular temperature during GFP. PL and LR were also increased from 6.2 to 6.9 and 5.8 to 6.5, respectively, under the heatstress. A boxplot analysis clearly showed the differential effect of heatstress on morpho-physiological traits in tolerant and susceptible accessions (Figure 5).

All the traits were reduced under the HS environment in both tolerant and susceptible accessions except CTD, PW and LR. However, mean values of CTD, PW and LR were higher under HS in the tolerant accessions. The tolerant accessions showed higher mean values for all traits as compared to heat-susceptible ones under the HS environment, except MSI. A higher MSI was observed in the susceptible accessions because of more leakage of cell contents at 100 °C and showed higher EC as compared to the tolerant accessions.

**Figure 6:**
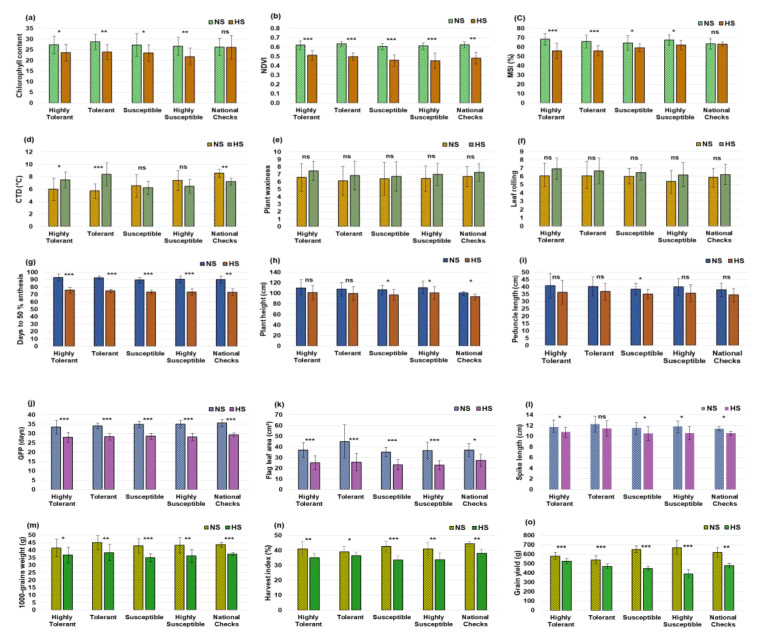
Effects of heatstress on important morpho-physiological and yieldtraits in tolerant and susceptible accessions of bread wheat. Reduction in CC and NDVI was more evident in highly susceptible and susceptible accessions compared to highly tolerant and tolerant accessions under HS environment (**a**,**b**). MSI was higher in highly susceptible and susceptible accessions than the tolerant accessions in HS environment (**c**). CTD, PW and LR increased in the tolerant accessions as compared to the susceptible ones under HS environment (**d**–**f**). Under heatstress, DA, PH and PL were reduced in both the tolerant and susceptible accessions (**g**–**i**). GFP, FLA and SL all decreased in both tolerant and susceptible accessions under heatstress (**j**–**l**). TGW, HI and GY were higher in the tolerant accessions than the susceptible ones in HS environment (**m**–**o**). Levels of significance (ns *p* ≥ 0.05, * *p* < 0.05; ** *p* < 0.01 and *** *p* < 0.001) were derived using *t* test.

The effects of heatstress on important traits were also investigated in five classes of wheat accessions, namely, highly tolerant, tolerant, susceptible and highly susceptible accessions, along with national checks. Under heatstress, the reduction in CC and NDVI was more evident in susceptible accessions as compared to tolerant. However, under the HS environment, MSI showed relatively higher values in susceptible accessions. CTD revealed a significant increase in tolerant accessions as compared to the susceptible ones under the HS environment (Figure 6). However, PW and LR showed higher values in tolerant accessions under heatstress but they were not significant when compared to the NS environment. DA, FLA, GFP, TGW, HI and GY were reduced significantly both in tolerant and susceptible accessions under heatstress. However, PH, PL and SL showed a non-significant reduction in tolerant accessions under the HS environment.

### 3.6. Impact of HeatStress on Wheat Grains

**Figure 7:**
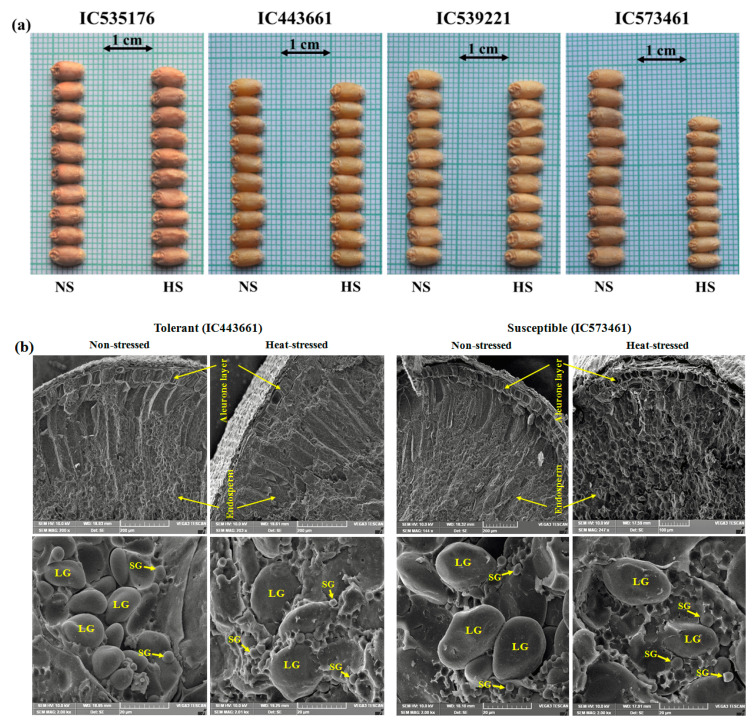
Impact of heatstress on grain morphology and ultrastructure as visualized with scanning electron microscopy (SEM) in tolerant and susceptible accessions of bread wheat. Reduction in grain width due to heatstress was more evident in susceptible accession IC573461 as compared to three tolerant accessions, IC535176, IC443661 and IC539221 (**a**). Ultrastructural changes caused by heatstress in endosperm and aleurone layer of grains in very-late-sown bread wheat accessions (**b**). SEM revealed ultrastructure of matured wheat grains, showing aleurone layer and endosperm ((**b**), upper panel; low magnification) and packing of starch granules (structure and density as seen in close-up view) in the endosperm ((**b**), lower panel; high magnification) in tolerant (IC443661) and susceptible (IC573461) accessions in non-stressed (NS) and heat-stressed (HS) environments.

Heatstress reduced GW, GL and TGW in all the accessions. However, the effect of heatstress on grain traits was more evident in susceptible than tolerant accessions (Figure 7). The reduction in GW was significantly high in the susceptible compared to tolerant accessions (Figure 7a). SEM analyses of the aleurone layer and endosperm were carried out in mature wheat grains of heat-tolerant and -susceptible accessions grown in NS and HS environments (Figure 7b). The heatstress during GFP adversely affected the aleurone layer and endosperm of wheat grains. The concentration and morphology of starch granules changed under heat stress. The size, shape and structure of the aleurone layer and starch granules of heat-stress-tolerant grains were quite different from the heat-stress-susceptible genotypes. The aleurone layer was destructured in the susceptible accessions (Figure 7b, upper panel).

The heatstress showed adverse effects on the structure and packing of starch granules. Robust, bold and well-structured large starch granules (LG) were observed in both the heat-stress-tolerant and -susceptible accessions under the NS environment, whereas unstructured, shriveled LG with a prominence of small starch granules (SG) were present in the wheat grains developed under the HS environment. The density of LG (A-type; 15–35 µm) was higher in both heat-stress-tolerant and -susceptible accessions in the NS environment, whereas, in the HS environment, the density of LG reduced slightly in tolerant accessions while it was considerably reduced in the susceptible accessions. The density of SG (B-type; 2–8 µm) was higher in both tolerant and susceptible accessions under the heatstress.

### 3.7. Selection of Heat-Stress-Adapted Germplasm

**Figure 8:**
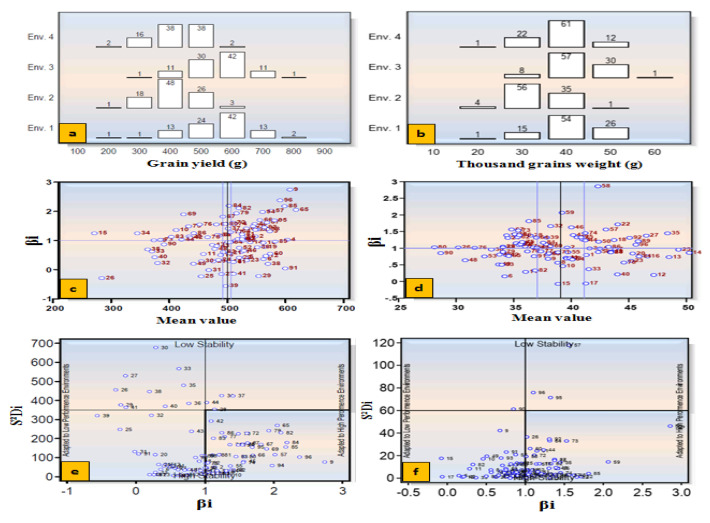
Stability parameters for GY and TGW based on Eberhart and Russell model. Frequency distribution (**a**,**b**), mean performance and regression value (β_i_) (**c**,**d**) and stability based on S^2^D_i_ (**e**,**f**) of 96 bread wheat accessions for GY and TGW.

Grain yield and TGW were used for a stability analysis using four environment datasets. ANOVA showed significant differences (*p* < 0.01) among accessions (=Genotype, G) and environments (E) for both the traits (Supplementary Table S5). The grain yield exhibited significant interactions for G × E, G × E (Linear), E + (G × E) and E (Linear). However, TGW revealed significant interactions for E + (G × E) and E (Linear), but non-significant interactions for G × E and G × E (Linear). On the basis of the mean performance, regression coefficient (βi) and HSI, wheat accessions were classified as adapted to the unfavorable (HS) environment, favorable (NS) environment or both environments (Table 3; Figure 8). Accessions IC566223, IC529207, IC335792, IC535176, EC576707, IC128454, IC416019, IC446713, IC265318, IC252348, IC401976, IC075240 and IC539531 performed well under the HS environment and were identified as highly tolerant genotypes. Accessions IC252431, IC277741, EC190899, CUO/79/Pru11A, EC277134, IC524299, IC553599, EC576585, IC573461 and EC576066 performed poorly in the HS environment, and are highly susceptible.

Five accessions, namely, IC393878, IC416018, IC539221, EC534487 and IC443661, performed well under both the environments, and are called general adapters. Likewise, stability for TGW revealed that some accessions were well adapted to HS, NS or both environments (Table 3, Figure 8).

The details of mean performance and stability parameters of 96 wheat accessions for TGW and grain yield are provided in Supplementary Table S6. The accessions showing a higher mean than the population mean, regression coefficient (β_i_) < 1 and HSI < 0.5 and producing >500 g of grain yield were considered as highly tolerant genotypes (Supplementary Table S7). The top 10 accessions were screened out for various morpho—physiological, yield and contributing traits under both NS and HS environments (Supplementary Table S8).

We selected germplasm lines based on their performance under the HS environment as potential parents for the development of bi-parent and MAGIC (Multi-Parent Advanced Generation Inter-Cross) populations (Table 4). Accessions, which showed extreme phenotypes under the HS environment, were chosen as potential parents for the creation of trait-specific bi-parent mapping populations. However, for the selection of promising parents for four-parent and eight-parent MAGIC populations, accessions showing the highest expression of yield and its associated morpho-physiological traits were considered.

## 4. Discussion

The production and productivity of bread wheat are adversely affected due to terminal-heat stress as a result of delayed sowing in many parts of India [21]. We evaluated a diverse set of 96 accessions of bread wheat to analyze the plant responses to heatstress, and their effects on morpho-physiological and yield-related traits. Proper endosperm development under heatstress is the key to maintain TGW, yield level and grain quality in wheat, and to facilitate the selection of superior and stable genotypes under an HS environment [36,51]. The heatstress imposed by sowing the wheat trial in a very late condition during the first week of January in both years created a unique environment to test germplasm for heat-stress tolerance. Delayed-sown wheat is exposed to higher temperatures at reproductive and grain filling stages, and this is a widely used strategy to screen germplasm for yield and other traits under heatstress [52,53]. Yield, a complex quantitative trait, is the endproduct of many interactions between genes for physiological and yield component traits. Heatstress has a wide range of effects on plants in terms of physiological, biochemical and gene regulation pathways [54].

### 4.1. Trait Variability and Impact of HeatStress

The wheat accessions showed a varied response to heatstress and provided an ample scope for the selection of trait-specific accessions. We observed a high level of variability in the germplasm accessions. A non-significant genotype × year (G × Y) interaction for most of the traits reveals that accessions had similar expressions in both the years. The significant G × Y interactions for NDVI in both environments; TGW under NS; and PH, PL, SL and GL under the HS environment suggest that accessions performed differently over the years for these traits. Our results corroborate earlier studies [35,55], which also reported varied responses of these traits and their interactions with the environment. The heat-adapted genotypes with the best yielding ability also possessed a high early biomass, high grain filling rates and a low canopy temperature [56].

The response to heat stress involves physiological adaptations, required to protect vital cellular functions like photosynthesis and homeostasis of metabolites [54,57]. The heatstress had an adverse effect on crop growth and development, and it consequently negatively affected morphological and yield-related traits, namely, DA, PH, PL, FLA, GFP, SL, NSS, HI, GL, GW, TGW and GY. Physiological traits, viz., CC, NDVI and MSI, were reduced, whereas CTD, PW and LR were enhanced, under the HS environment. The reduction in the above traits was more prominent in the susceptible accessions. Such a reduction in morphological and yield traits was also reported in earlier studies [28,36,37,53]. A CTD analysis is a reliable and non-invasive method for selecting heat-tolerant lines [58]. Like this study, a reduction in days to heading, GFP, PH, HI, TGW and GY under heatstress was also observed earlier [28,36]. The extent of reduction for a given trait varied in these studies due to the use of a different set of genotypes and test conditions. The yield and its component traits are more severely affected with the increase in heat stress. The yield parameters were also reduced in the plants exposed to heatstress in wheat landraces [59]. Recently, in India, areduction in DA, MSI and yield traits was reported in late-sown wheat [60]. This study also observed that HSI was the lowest in the heat-tolerant germplasm and supports our observations. A higher leaf waxiness under an HS condition was also reported earlier [25], supporting our results of an increased waxiness under heatstress, which protects a plant against excess radiation and water loss through the reflection of visible and infrared wave lengths [61]. The heritability of waxiness is low because of significant G × E interactions [62]. Genotypes with the stay-green trait performed better under heat stress and could be used for heat stress tolerance breeding [63].

### 4.2. Association of Grain Yield with Other Traits

Grain yield, a complex quantitative trait, is controlled by many interactions between morphological, physiological and related parameters. Under the HS environment, the yield showed positive correlations with GFP, HI, GW, PW and LR. The positive correlation of yield with GFP reveals that a longer grain development period is a vital factor for improving yield under heatstress. In a previous study [36], grain yield and HI showed a positive correlation with TGW under both normal and terminal-heat stress conditions, whereas GFP was positively associated with TGW only under the heatstress. This study advocates that the selection for a low TGW reduction is an indirect criterion to identify high yielding lines under terminal heatstress. In our study, TGW revealed an association with yield only under the NS environment. However, another study [64] reported an association of TGW with yield under both optimal and HS environments. A positive association of yield with GFP and TGW was also reported under heatstress [65]. There was a non-significant association of grain yield with DA, PH and TGW in the present study under the HS environment, which confirms the results of an earlier study [53]. Hence, the focus should be on the traits showing a high association with yield under the HS environment for the selection of heat-tolerant lines.

In our study, CC did not show an association with GY, but CTD revealed a significant association with GY only under the heatstress. Similarly, Elbasyoni [35] also observed no correlation between CC and GY. However, a high correlation of both CTD and CC with GY under heatstress was reported earlier [24,66]. High genetic variations for CC and TGW along with a positive association between these two traits were found under a heat- stress condition in elite winter wheat lines [67]. Similarly, an association of CTD with GY was also observed under both NS and HS environments [23]. Under heatstress, GY displayed a strong positive correlation with both CTD and days to heading [68,69,70]. The longer time before heading enables the development of large spikes with extra spikelets producing more grains per plant. The association studies reveal that grain yield in wheat correlates positively with some physiological and yield-related traits under heat stress. Thus, the selection of these traits could be vital for heat-stress-tolerance breeding.

### 4.3. Grain Development under Heat Stress

Heat stress during GFP adversely affects the grain size in bread wheat. In our study, the effect of heatstress on GW was more severe as compared to GL, which resulted in shriveled grains in susceptible accessions. The development of shriveled grains in the susceptible accessions was due to changes in the ultrastructure of aleurone cells and starch granules in endosperm. Our findings on grain development under heatstress corroborate a recent study [71], wherein they observed a severe effect of heatstress on grain traits like GL, GW and grain area along with starch synthesis. The effect of heatstress shows a significantly reduced GW and perimeter. The endosperm of mature wheat grain contains two types of starch granules: large (10–35 µm) A-type and small (1–10 µm) B-type [72,73]. The density and size of the large type of starch granules were slightly reduced in the tolerant accessions while these were reduced considerably in the susceptible accessions under the HS environment. The concentration of small starch granules was higher in both the heat-tolerant and -susceptible accessions under the HS environment. Our findings on the size and density of starch granules are supported by a previous study [74], which reported that the ratio of large and small starch granules decreases significantly under heatstress and this limits the potential sink size for dry matter deposition in wheat grains. Heat stress during GFP triggers ultrastructural changes in the aleurone layer and endosperm, and causes disordered cells, grain shrinkage and a reduced TGW in susceptible genotypes [31].

Grain development is influenced by various metabolic processes occurring in leaves, mainly the production and translocation of photoassimilates and importing of precursors for the biosynthesis of grain reserves, minerals and other functional constituents [75]. Hence, it is crucial to know the physiological, biochemical and genetic mechanisms that govern the grain development events under heatstress to devise strategies for yield enhancement in wheat. The identification and selection of germplasm lines with a higher yield and grain weight along with an early maturity, semi-tall plant height, higher NDVI during GFP and higher CC at the milky stage ought to be the hallmark of breeding strategies for heat-stress tolerance [53,64]. Harnessing the genetic variability for these traits in the germplasm is vital for the breeding of heat-stress-tolerant cultivars.

### 4.4. Yield Stability and Selection of Heat-Adapted Accessions

Heat-stress adaptation is a complex phenomenon and is influenced by several factors such as the genotype and its interaction with the environment over a long period of time. A wheat plant achieves adaptation through variation in phenology and other related traits determining the plant architecture [42]. Hence, it is crucial to understand genes that underpin the variations in plant phenology and physiology, and their interactions with other genes and the environment. The yield stability across the environments is a reliable criterion for the selection of heat-stress-adapted germplasm [41,76]. Climate-change- associated global warming has severely affected yield stability in cereal crops [77]. G×E interactions are of major importance to a plant breeder for developing improved varieties. We used a stability analysis [50] to identify stable and better yielding accessions under heatstress. The better-performing accessions in the HS environment might be used as donor parents for a heat-stress-adaptation breeding program for the IGP region. A similar study was carried out to select superior yielding lines under heatstress [35]. However, G×E interaction biplots were used to select genotypes with a stable performance across all environments [19,29]. A selection strategy was also suggested to improve adaptation to heat stress in bread wheat [53]. Heat stress significantly affects all the yield contributing traits and TGW, an important trait for the selection of tolerant lines. The heat-tolerant lines with a high grain yield could be selected using HSI and stability analyses [78]. The stable and higher yielding accessions identified under the HS environment could be utilized in breeding programs for the development of heatstress-tolerant wheat cultivars.

## 5. Conclusions

Wheat genetic resources used in the present study represent a part of the reference set for heat-stress tolerance and hence exhibited a high extent of genetic variability for morpho-physiological and yield-related traits. The terminal-heatstress in the late-sown bread wheat crop negatively affected grain yield and its contributing traits. The higher temperature than the optimum temperature during GFP reduced the grain size, and eventually decreased TGW and grain yield under the HS environment. The ultrastructural analysis of matured grains showed that the decrease in size and density of large starch granules in endosperm is the main cause of the yield and TGW reduction in the heat- stress-susceptible accessions. The high yielding accessions possessing desirable morpho- physiological traits and adaptation to the HS environment were identified and selected, which could be utilized for the development of mapping populations and the genetic improvement of bread wheat for heat tolerance.

## Figures and Tables

**Table 1:** Morpho-physiological and yield-related traits studied in 96 bread wheat accessions under non-stressed and heat-stressed environments during two crop seasons of 2018–2019 and 2019–2020.

S. No.	Traits Studied	Code	How Was the Trait Measured?
1.	Chlorophyll Content	CC	Estimated on flag leaves of five random main tillers in each accession with hand-held Chlorophyll Content Meter (Model-CCM-200 plus, Opti-Sciences, Hudson, NH, USA).
2.	Canopy Temperature Depression (°C)	CTD	Measured on warm, sunny and clear day using portable Infrared Thermometer (Fisher Scientific, Loughborough, Leicestershire, UK).
3.	Normalized Difference Vegetation Index	NDVI	NDVI was recorded using hand-held crop sensor (Green Seeker^®^, Trimble, Westminster, CO, USA). It ranged from 0 to 1; 0 refers to no green area and 1 to maximum greenness.
4.	Membrane Stability Index (%)	MSI	MSI was estimated with small leaf discs of uniform size cut from 0.1 g leaf samples of each wheat accession and calculated using the following formula: MSI = [1 − (C1/C2)] × 100, where C1 and C2 represent readings of EC (Electrical Conductivity) recorded using digital conductivity meter at 45 °C and 100 °C, respectively.
5.	Days to 50% Anthesis	DA	Recorded as the period between the date of sowing and the dateat which 50% of spikes start to extrude their anthers.
6.	Grain Filling Period (days)	GFP	GFP calculated as the difference between days to 50% anthesis and days to physiological maturity.
7.	Plant Waxiness (0–10 scale)	PW	PW was measured with visual observations of whole plot during mid of GFP and scored using scale from 0 (0%) to 10 (100%) in an increment of 10%.
8.	Leaf Rolling (0–10 scale)	LR	LR was measured at mid of GFP with visual observation of whole plot and scored as proportion of the leaves showing rolling effect using a scale from 0 (0%) to 10 (100%) in an increment of 10%.
9.	Plant Height (cm)	PH	PH was measured from base of the plant to top of the spike excluding awns of the main tiller at maturity.
10.	Peduncle Length (cm)	PL	Measured from uppermost node to the spike collar of the main tiller at maturity in three plants per accession.
11.	Flag Leaf Area (cm^2^)	FLA	Derived from five randomly chosen plants’ flag leaves using the equation Leaf area = Length × Breadth × 0.75.
12.	Spike Length (cm)	SL	Measured from the spike collar to tip of the spike excluding awns of the main tiller in three plants per accession.
13.	Number of Spikelets per Spike	NSS	Spikelets per spike were counted on the main tiller spike of three plants per accession.
14.	Grain Length (mm)	GL	Measured on five grains per accession with Digimatic Caliper (Model-CD-6″ASX, Mitutoyo Corporation, Kawasaki, Kanagawa, Japan).
15.	Grain Width (mm)	GW	Grain width was measured from five grains randomly selected per accession with Digimatic Caliper.
16.	1000-Grain Weight (g)	TGW	TGW was recorded from 1000 grains randomly selected from plot yield and weighted using sensitive electronic balance (d = 0.1 mg, Sartorius, model CPA64, Göttingen, Lower Saxony, Germany).
17.	Harvest Index (%)	HI	HI was calculated using the following formula: HI = (Grain yield per plant/Biological yield per plant) ×100.
18.	Grain Yield (g/m^2^)	GY	Plot yield of each accession harvested, threshed manually, and weight of grains recorded with electronic balance. GY is expressed as yield per unit area.

**Table 2:** Descriptive statistics of pooled data of 18 morpho-physiological and yield traits recorded in 96 accessions of bread wheat in NS and HS environments during two crop seasons of 2018–2020.

Trait	Environment	Range		Mean ± S.E.	SD	CV (%)	PCV (%)	GCV (%)	H^2^ (%)	GA (%)
		Min.	Max.							
CCI	NS	17.4	38.5	26.9 ± 0.43	4.21	15.69	18.1	15.0	68.3	25.5
	HS	13.5	37.2	22.7 ± 0.49	4.10	18.10	18.6	16.2	75.9	29.0
CTD (°C)	NS	2.6	9.7	6.2 ± 0.18	1.80	29.19	23.3	10.5	20.3	9.7
	HS	4.4	12.0	6.9 ± 0.15	1.47	21.24	26.1	11.0	17.8	9.6
NDVI (0–1)	NS	0.54	0.72	0.62 ± 0.01	0.04	6.03	7.5	5.1	42.9	6.6
	HS	0.32	0.64	0.48 ± 0.01	0.06	12.61	10.9	8.5	63.8	14.3
MSI (%)	NS	50.0	77.5	66.6 ± 0.70	6.89	10.35	10.5	6.0	33.0	7.2
	HS	43.7	72.6	59.4 ± 0.68	6.70	11.29	8.5	2.5	8.7	1.5
PW (0–10)	NS	1.0	10.0	6.2 ± 0.20	1.94	31.35	23.8	19.6	67.6	33.2
	HS	2.0	10.0	6.9 ± 0.17	1.68	24.47	21.4	17.2	64.6	28.5
LR (0–10)	NS	2.5	9.5	5.8 ± 0.13	1.28	22.04	20.8	17.5	71.4	30.5
	HS	4.0	10.0	6.5 ± 0.13	1.26	19.29	19.1	15.0	61.4	24.1
Days to 50% anthesis	NS	83.4	119.0	91.9 ± 0.48	4.72	5.13	6.1	5.6	84.9	10.6
	HS	66.6	94.0	74.6 ± 0.40	3.88	5.20	6.8	6.3	85.4	11.9
GFP (days)	NS	24.0	39.5	34.2 ± 0.25	2.45	7.16	19.9	10.7	29.0	11.9
	HS	23.5	32.0	28.1 ± 0.20	1.94	6.91	8.7	5.7	43.0	7.7
Plant height (cm)	NS	84.9	150.9	106.6 ± 1.31	12.83	12.03	5.9	2.5	17.8	2.2
	HS	73.2	127.0	96.9 ± 1.25	12.23	12.63	7.1	4.4	39.1	5.7
Peduncle length (cm)	NS	29.4	60.1	39.2 ± 0.61	6.00	15.33	14.7	12.1	67.7	20.6
	HS	27.0	53.8	35.1 ± 0.54	5.27	15.04	14.5	12.9	79.4	23.7
Flag leaf area (cm^2^)	NS	21.9	77.0	37.4 ± 0.88	8.61	23.01	20.4	16.1	62.4	26.2
	HS	14.2	51.7	23.8 ± 0.61	5.95	24.96	17.8	14.5	66.3	24.3
Spike length (cm)	NS	8.7	15.6	11.7 ± 0.12	1.18	10.14	6.8	3.0	19.4	2.7
	HS	8.1	13.5	10.6 ± 0.11	1.10	10.38	4.8	1.3	7.1	0.7
Spikelets per spike	NS	16.0	24.0	20.2 ± 0.15	1.46	7.23	5.9	2.1	12.7	1.6
	HS	15.5	22.7	18.7 ± 0.15	1.49	7.98	5.9	2.4	16.3	2.0
Grain length (mm)	NS	5.97	8.76	6.90 ± 0.04	0.39	5.59	5.6	4.9	77.9	9.0
	HS	5.85	8.62	6.68 ± 0.04	0.38	5.63	6.1	5.8	89.3	11.3
Grain width (mm)	NS	2.84	4.02	3.49 ± 0.02	0.19	5.31	3.5	2.0	33.0	2.4
	HS	2.64	3.68	3.27 ± 0.02	0.18	5.52	3.3	1.4	17.9	1.2
1000-grain weight (g)	NS	30.0	52.2	41.5 ± 0.53	5.19	12.51	6.0	3.0	24.1	3.0
	HS	24.9	46.6	35.5 ± 0.48	4.73	13.34	8.0	3.3	16.7	2.8
Harvest index (%)	NS	21.6	50.6	39.7 ± 0.49	4.84	12.19	9.4	1.7	13.3	0.6
	HS	23.4	48.0	34.4 ± 0.45	4.38	12.76	9.3	3.2	11.9	2.3
Grain yield (g/m^2^)	NS	300.0	802.5	562.2 ± 8.77	86.00	15.30	11.3	7.3	42.1	9.8
	HS	176.7	598.1	423.6 ± 7.34	71.95	16.97	10.5	5.8	30.2	6.6

**Table 3:** Grouping of different wheat accessions based on Eberhart and Russell stability model and their mean performance under NS and HS environments for TGW and GY along with HSI.

Adaptation	Wheat Accession	TGW			Grain Yield			TGW		Grain Yield		HSI
		µ	β_i_	S^2^D_i_	µ	β_i_	S^2^D_i_	NS	HS	NS	HS	
**Unfavorable (heat-stressed) environment**	IC543425	37.4	1.32	0.09	487.2	−0.56	319.85	39.7	31.9	508.0	498.0	0.08
	IC128454	33.6	1.05	1.91	543.6	−0.22	376.52	34.9	29.1	546.0	538.1	0.05
	IC265318	39.1	0.71	11.30	500.7	−0.15	363.40	38.5	36.4	508.0	504.1	0.03
	IC252348	46.0	1.38	16.61	480.1	−0.15	529.35	46.9	41.9	488.0	476.7	0.09
	IC566223	36.3	0.76	23.13	589.5	0.04	119.09	40.4	34.7	604.3	598.1	0.04
	IC335792	33.6	1.02	7.37	559.7	0.20	446.45	34.0	29.9	593.4	539.4	0.37
	IC290191	37.3	1.12	1.93	500.5	0.26	9.76	39.7	34.5	522.2	482.7	0.31
	IC401976	48.0	0.73	4.67	493.3	0.28	21.89	49.7	45.2	510.5	478.1	0.26
	IC446713	44.7	0.62	1.02	522.7	0.32	11.41	46.6	41.7	545.2	505.4	0.30
	EC576707	33.7	0.15	2.20	552.1	0.36	37.58	33.8	32.4	579.2	534.7	0.37
	IC075240	42.9	0.88	6.98	474.5	0.33	63.50	44.7	39.9	501.9	460.1	0.34
	IC416019	43.6	1.71	1.29	510.0	0.41	55.05	49.1	36.8	542.5	490.1	0.40
	EC574731	42.0	0.99	0.22	520.7	0.43	62.51	45.2	37.6	549.2	500.7	0.36
	IC535176	46.7	0.19	0.94	556.0	0.47	61.91	47.1	45.0	593.9	532.1	0.43
	IC539531	46.1	0.75	2.11	478.3	0.50	49.48	48.5	42.6	517.2	452.1	0.52
	IC529207	37.7	1.06	26.18	563.5	0.55	5.10	39.3	35.7	604.9	527.4	0.52
**Both environments**	IC416018	43.0	0.89	13.29	553.8	0.78	36.99	47.1	37.6	605.9	508.1	0.66
	IC443661	40.8	−0.06	1.18	463.9	0.85	24.38	41.1	39.2	523.2	413.4	0.86
	EC534487	43.2	1.24	2.62	551.6	0.92	80.85	47.7	37.6	609.9	496.1	0.76
	IC539221	49.8	0.86	3.33	529.0	0.94	12.34	51.8	46.6	593.2	468.7	0.86
	IC393878	45.3	1.21	3.85	572.2	0.96	109.62	49.3	43.9	633.7	516.7	0.75
**Favorable (non-stressed) environment**	IC573461	41.8	2.86	46.15	558.5	1.39	34.86	52.2	31.1	654.9	462.7	1.20
	IC535717	39.4	0.69	17.82	553.6	1.45	104.97	43.4	38.0	649.0	464.1	1.17
	IC144911	33.3	0.81	0.05	537.0	1.56	79.27	35.0	31.1	648.9	425.4	1.41
	EC576175	40.0	1.32	3.41	557.9	1.62	176.60	44.4	38.1	661.7	456.7	1.27
	EC277134	40.4	1.32	71.39	570.8	1.69	154.00	48.0	35.3	680.4	464.7	1.30
	IC252619	35.0	1.39	1.74	545.9	1.70	112.16	40.2	32.2	659.5	441.7	1.35
	IC529242	34.3	1.56	2.87	482.2	1.82	169.29	40.5	30.7	600.9	369.1	1.58
	IC443694	33.6	0.89	5.34	505.4	1.93	240.81	37.7	31.9	630.9	387.7	1.58
	IC553599	39.6	0.66	2.56	550.5	1.98	57.60	42.4	39.3	685.7	424.7	1.56
	IC524299	42.5	1.56	117.81	565.8	2.02	114.97	50.5	34.1	709.5	422.7	1.65
	IC252431	34.1	0.76	1.61	607.9	2.05	269.05	37.7	33.0	739.5	478.4	1.44
	EC190899	35.8	1.81	3.98	588.3	2.18	155.19	41.1	33.1	732.4	448.7	1.58
	EC576585	45.4	0.75	7.26	492.9	2.19	178.97	48.5	44.7	637.5	353.7	1.82
	CUO/79/Pru 11A	45.1	1.10	75.79	580.5	2.38	102.43	52.1	40.7	741.0	423.4	1.75
	IC277741	37.5	0.68	42.16	597.6	2.74	76.59	38.0	35.8	802.5	416.1	1.97
**Nationalchecks**	RAJ3765	40.8	0.80	4.03	514.2	0.61	35.67	43.0	38.7	552.5	472.0	0.60
	HD2932	39.2	1.02	7.19	538.1	1.14	49.90	41.7	36.6	614.7	458.8	1.04
	WR544	41.1	0.99	3.10	562.8	1.31	25.24	44.6	37.7	655.1	472.1	1.14
	HD2967	40.5	1.25	3.38	592.7	1.03	46.12	44.9	36.2	665.2	518.7	0.90
Population mean		38.6	-	-	489.4	-	-	41.5	41.5	562.2	423.6	1.00
LSD (5%)		-	-	-	-	-	-	2.1	2.6	49.0	21.1	-

Abbreviations: TGW: 1000-Grain weight, HSI: Heat susceptibility index, NS: Non-stressed environment, HS: Heat-stressed environment, μ: Mean, β_i_: Regression coefficient, S^2^d_i_: Deviations from the regression.

**Table 4:** Identification and selection of parents for the development of mapping populations for heat-stress tolerance in bread wheat.

Sl. No.	Traits for Mapping Population	Parents with Desirable Traits for Heat-Stress Tolerance	
**(a).**	**Bi-Parent Population**	**Parent (Higher Value)**	**Parent (Lower Value)**
1.	Plant waxiness	IC529207, IC528965	IC252431, IC252444
2.	Leaf rolling	IC416019, IC416055	IC553599, IC252816
3.	Earliness	IC296383	IC542509
4.	Grain filling period	IC252725	EC577013
5.	Grain width	IC401976	IC112258
6.	1000-grain weight	IC539221	IC542544
7.	Harvest index	IC443653	IC542509
8.	Grain yield	IC566223	EC577013
**(b).**	**MAGIC Population**	**Parents with Desirable Traits**	
1.	4-parent MAGIC	IC566223, IC529207, IC416019, IC296383	
2.	8-parent MAGIC	IC128454, IC519900, IC528965, IC416055,	
		IC539221, IC401976, IC535176, IC566223	

## Data Availability

The original contributions are included in the article/supplementary files. Further queries can be directed to the corresponding author.

## Supplementary Materials

The following supporting information can be downloaded at: https://www.mdpi.com/article/10.3390/plants12152851/s1, Figure S1: Geo-referencing map of bread wheat accessions, Figure S2: Frequency distribution of 96 bread wheat accessions for 12 important morpho-physiological and yield contributing traits, Table S1: Passport details of 96 bread wheat accessions, Table S2: ANOVA for combined ABD data of 18 traits under NS and HS environments, Tables S3 and S4: Combined adjusted mean values for 18 traits under NS and HS environment, respectively, Table S5: ANOVA for TGW and GY based on stability analysis, Table S6: Mean performance and stability parameters for TGW and GY, Table S7: Identified heat-stress-tolerant and -susceptible wheat accessions, Table S8: Promising accessions selected for different traits for NS and HS environments.
