# Supplementary material for: Identification of Climate-Smart Bread Wheat Germplasm Lines with Enhanced Adaptation to Global Warming
Source: Plants (Basel). 2023 Aug 2;12(15):2851. doi: 10.3390/plants12152851 (PMC10420658; doi:10.3390/plants12152851)
Supplement: Supplementary file 1 [file plants-12-02851-s001.zip › plants-2450699-supplementary.pdf]

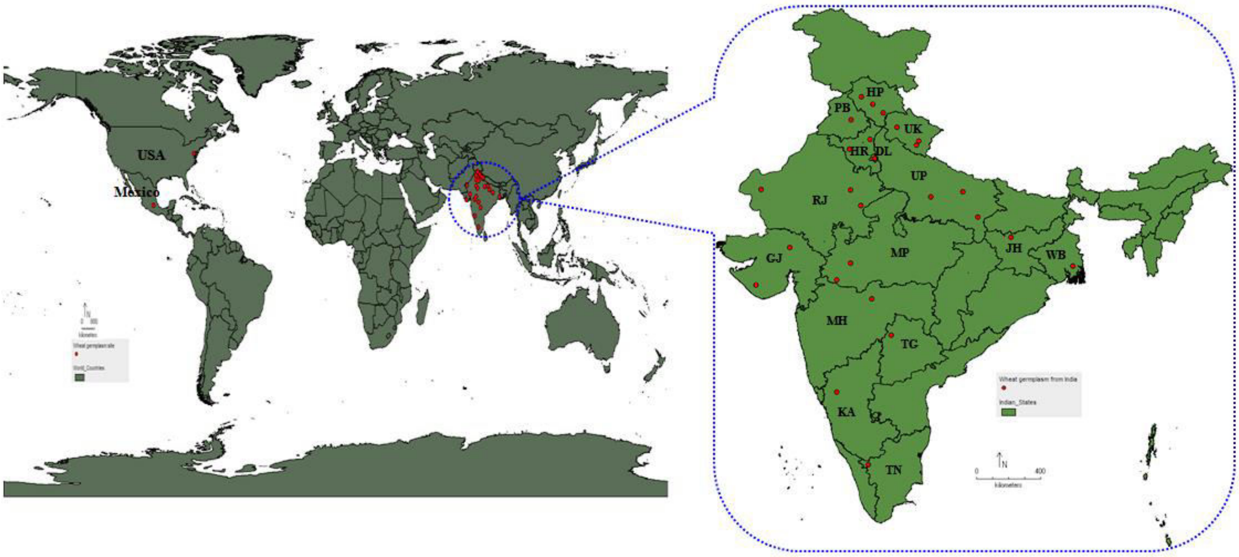

Supplementary Figure S1│ Geo-referencing map of bread wheat accessions comprising of exotic (17) and indigenous (79) collections used in the screening for terminal heat-stress tolerance.

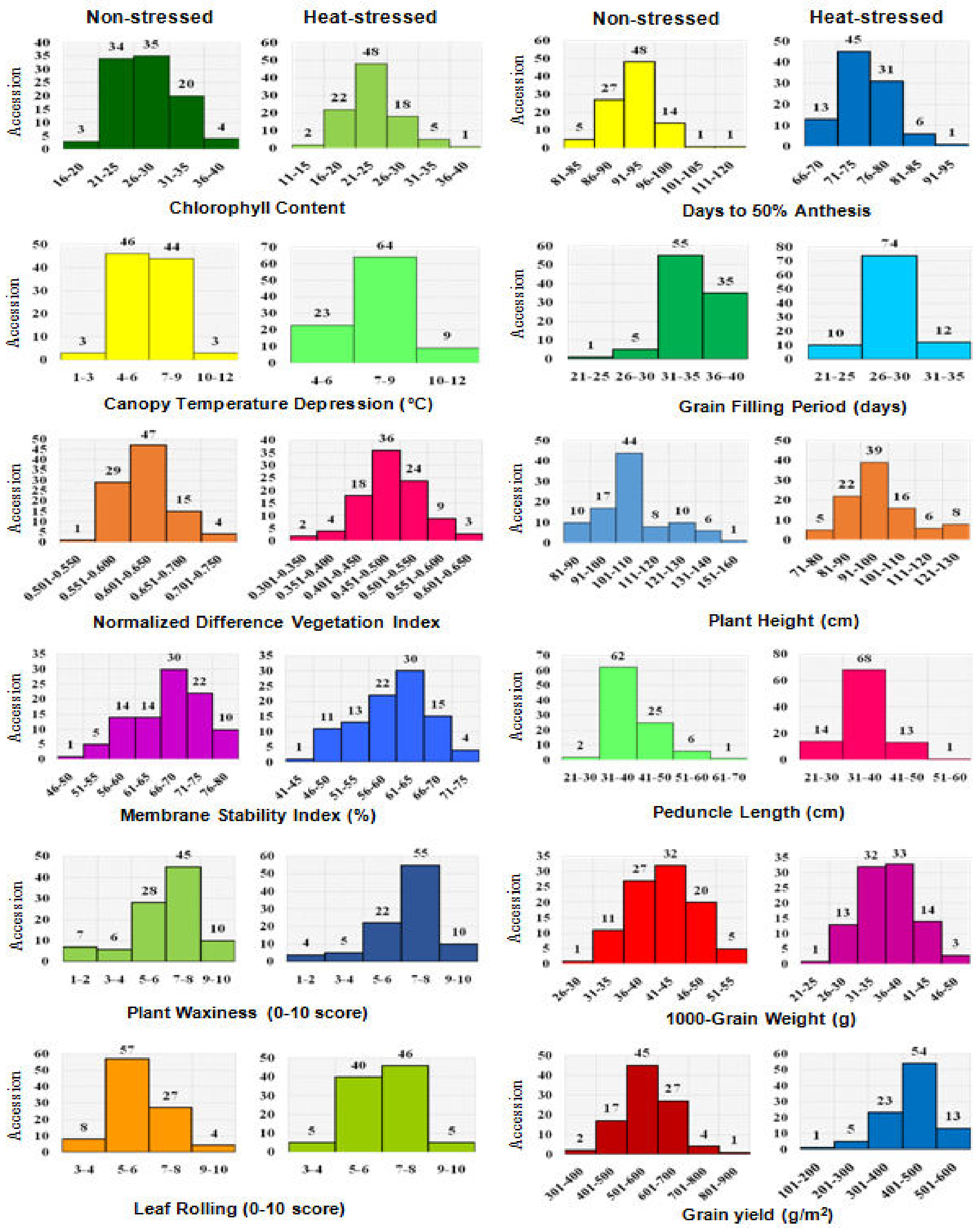

Supplementary Figure S2 | Frequency distribution of 96 bread wheat accessions for 12 important morpho-physiological and yield contributing traits under non-stressed and heat-stressed environments.

Supplementary Table S1 │ Details of bread wheat accessions used in the study along with their IC/EC (Indigenous/Exotic Collection) number, alternate identity and original source of acquisition.

Sl. No.	Accession Number	Alternate id	Original Source	Year of Release/ Collection	Parentage	Remarks	Recommended for cultivation (ecology and zone)
1.	IC443766	RAJ3765	RARI, Jaipur, Rajasthan	1996	HD 2402/VL639	Tolerance to terminal heat-stress	Irrigated, late sown conditions of CZ
2.	IC519900	HD2932	IARI, New Delhi	2008	KAUZ/STAR// HD 2643	Brown rust resistance and high zinc content	Irrigated, late sown conditions of CZ
3.	IC296383	WR544 (Pusa Gold)	IARI, New Delhi	2005	KALYANSONA/HD 1999//HD2204/DW38	Very early maturing, resistance to brown rust	Irrigated, late/very late sown, for Delhi state
4.	IC574476	HD2967	IARI, New Delhi	2011	ALD/COC//URES/ HD2160M/HD2278	Wider adaptability and resistance to yellow and brown rust	Irrigated, timely sown conditions of NWPZ
5.	EC574731	8832	CIMMYT, Mexico	-	-	Germplasm line	-
6.	EC576707	E4828	CIMMYT, Mexico	-	-	Elite line/germplasm	-
7.	IC252725	HUW467	BHU, Varanasi	1999	-	Germplasm collection	Irrigated, Timely sown, NEPZ
8.	IC252816	KRL13	IIWBR, Karnal	1999	-	Germplasm line with salinity tolerance	-
9.	IC277741	Dharwad 680	Dharwad, Karnataka	2000	-	Germplasm collection	-
10.	IC536081	WL3226	PAU, Ludhiana	-	-	Germplasm line	-
11.	IC279617	KHH416	Tehri, Uttarakhand	2000	-	Germplasm collection (Safed Gehun)	-
12.	IC535176	WON-D14	IARI, New Delhi	-	-	Germplasm line	-
13.	IC401976	PHR1011	IIWBR, Karnal	-	-	Germplasm line with High protein (>13%) and high TGW (>40 g)	-
14.	IC539221	EIGN-I-(04-05) /149	IIWBR, Karnal	2005	-	Germplasm collection	-
15.	IC539287	MC2003-71	IIWBR, Karnal	-	-	Germplasm line	-
16.	IC539531	EIGN1 (03-04)/112	IIWBR, Karnal	-	-	Germplasm line	-
17.	IC443661	PHR1017	IIWBR, Karnal	-	-	Germplasm line	-
18.	EC534487	VEE/KOEL (PAU351)	USDA, USA	-	-	Germplasm line	-
19.	IC416018	PAU438 (W 7484)	PAU, Ludhiana	-	-	Germplasm line	-
20.	IC416075	PAU495 (W 8067)	PAU, Ludhiana	-	-	Germplasm line	-
21.	IC416078	PAU498 (W 8093)	PAU, Ludhiana	-	-	Germplasm line	-
22.	IC416019	PAU439 (W 7485)	PAU, Ludhiana	-	-	Germplasm line	-
23.	IC446713	PSR11489 (Godhumalu)	Nizamabad, Telangana	-	-	Germplasm line	-
24.	IC075240	C306	CCSHAU, Hisar	1969	RGN/CSK3//2*C591/ 3/C217/N14//C281	Good for *chapatti* making quality	Rainfed, timely sown conditions of NWPZ
25.	EC178071	NA	CIMMYT, Mexico	-	-	Germplasm line	-
26.	IC542509	PAU1453	PAU, Ludhiana	-	-	Germplasm line	-
27.	IC252348	AKW2294	PDKV, Akola	-	-	Germplasm line	-
28.	IC543293	PAU1218	PAU, Ludhiana	-	-	Germplasm line	-
29.	IC128454	HB602	Bhowali, Uttarakhand	-	-	Germplasm line	-
30.	IC416055	PAU475 (W 7883)	PAU, Ludhiana	-	-	Germplasm line	-

31.	IC111800	J1-7	JAU, Junagadh	1975	Selection from J1	Pink colour auricle and dark brown ear colour	Restricted irrigation, timely sown for CZ
32.	IC111931	K101 (Kundan)	CSUAT, Kanpur	1969	HYBRID65/ HYBRID 1-1	-	Irrigated, timely/late sown, NEPZ
33.	EC576317	E1804	CIMMYT, Mexico	-	-	Elite line/germplasm	-
34.	EC577013	E8826	CIMMYT, Mexico	-	-	Elite line/germplasm	-
35.	EC414149	CHNQIRR 95S OCHN	CIMMYT, Mexico	-	-	Elite line/germplasm	-
36.	IC252653	HI1433	IARI RS, Indore	1999	-	Elite germplasm	-
37.	IC252739	HW1058	Wellington, TN	-	-	Elite germplasm	-
38.	IC335792	KAUZ+ 1B.1R	IIWBR, Karnal	-	-	Germplasm line	-
39.	IC543425	10TH HTW YT38	IARI, New Delhi	-	-	Elite line for heat tolerance	-
40.	IC402055	K20008 (KRL35)	IIWBR, Karnal	2004	-	Elite germplasm	-
41.	IC265318	NKD2671	Sawai Madhopur, Rajasthan	-	-	Germplasm collection (Katha Gehun)	-
42.	IC445449	ET99745	IIWBR, Karnal	-	-	Germplasm line	-
43.	IC528965	VWFW182	VPKAS, Almora	-	-	Germplasm line	-
44.	IC549437	PHR1032	IIWBR, Karnal	-	-	Germplasm line	-
45.	IC144911	VW120 (GW120)	RARS, Vijapur Gujarat	1985	INIA66/CNO//INI66/BB/3/Y50E/3*KAL	–	Irrigated, late sown conditions of NWPZ
46.	IC542578	PAU558	PAU, Ludhiana	-	-	Germplasm line	-
47.	IC535704	PBW124	PAU, Ludhiana	-	-	Elite germplasm	-
48.	EC542533	NA*	USDA, USA	-	-	Elite germplasm	-
49.	IC542652	PAU4061	PAU, Ludhiana	-	-	Germplasm line (Brochis susceptible)	-
50.	IC536468	K8415	CSUAT, Kanpur	-	-	Germplasm line	-
51.	IC536483	CPAN2039	IIWBR, Karnal	-	-	Germplasm line	-
52.	EC574735	8842	CIMMYT, Mexico	-	-	Germplasm line	-
53.	IC531191	PBW103	PAU, Ludhiana	-	-	Elite germplasm	-
54.	IC333095	NKD/YS R2910	Barwani, MP	-	-	Germplasm line (DL-803)	-
55.	IC572925	HPW185	Malan, Kangra (HP)	2009	-	Germplasm collection	-
56.	IC252867	NW1014	NDUAT, Ayodhya	1998	HAHN 'S'	High resistance against all the three rusts, leaf blight and Karnal bunt	Irrigated, late sown conditions of NEPZ
57.	IC524299	HW2012	IARI RS, Wellington	-	-	Elite germplasm	-
58.	IC573461	GW11 (GW396)	RARS, Vijapur, Gujarat	2013	LOK1/HW 1042// LOK1	High WUE variety and resistance to brown and black rusts	Gujarat state
59.	IC252444	BW/SH49	Kolkata, WB	1999	-	Germplasm collection	-
60.	IC529207	VWFW2150	VPKAS, Almora	2005	-	Germplasm collection	-
61.	IC290191	HW971	Wellington, TN	2000	-	Germplasm collection	-
62.	IC112258	VL401	VPKAS, Almora	1978	FKN/N 10B	-	Timely sown, rain-fed for U.P. hills
63.	IC627711	CAZ/JSM/ AP/01	Jaisalmer, Rajasthan	2018	-	Registered germplasm for conserved moisture	Cultivated in conserved moisture of Khading, Raj.
64.	IC443653	HD2851 (Pusa Vishesh)	IARI, New Delhi	2005	CPAN3004/WR426 // HW 2007	Resistance to yellow, brown and black rusts	Timely sown, irrigated for Delhi state
65.	IC252431	BW/SH30	Kolkata, WB	-	-	Germplasm line	-
66.	IC252619	HD2590	IARI, New Delhi	1999	-	Elite germplasm	-

67.	IC529242	VWFW1290	VPKAS, Almora	2005	-	Germplasm collection	-
68.	IC536162	WL4996	PAU, Ludhiana	-	-	Germplasm line	-
69.	IC536050	WL1803	PAU, Ludhiana	-	-	Germplasm line	-
70.	IC252999	WH594	CCSHAU, Hisar	1999	-	Germplasm collection	-
71.	IC443640	DWR240	IIWBR, Karnal	2004	-	Germplasm line with early maturity	-
72.	IC445365	7thEGPSN78	IIWBR, Karnal	2004	-	Elite line	-
73.	IC303071	RAJ3777	RARI, Jaipur	2006	RAJ3160/HD2449	Tolerance to terminal heat-stress	Late sown, Irrigated/ Rainfed for HP
74.	IC252414	BW1050	Kolkata, WB	1999	-	Germplasm collection	-
75.	IC372643	KRR/AK7	Mandi, HP	2002	-	Germplasm collection (Kanak)	-
76.	IC252620	HD2615	IARI, New Delhi	1999	-	Germplasm line	-
77.	IC240818	GW273	RARS, Vijapur, Gujarat	1998	CPAN 2084/VW205	Field resistance to brown and black rusts, high sedimentation value	Irrigated, timely sown conditions of CZ
78.	IC401940	K8962 (Indra)	CSUAT, Kanpur	1996	K7401/HD 2160	Tolerance to brown and black rust	Rainfed, late sown conditions of NEPZ
79.	IC443694	PBW527	PAU, Ludhiana	2008	PBW 175/PBW389	Resistance to yellow and brown rust	Timely sown, rain-fed for Punjab state
80.	IC542547	PAU4091	PAU, Ludhiana	-	-	Germplasm line	-
81.	EC190962	Cultivar No.30	CIMMYT, Mexico	-	-	Elite line/germplasm	-
82.	EC576066	410	CIMMYT, Mexico	-	-	Elite line/germplasm	-
83.	EC573527	3850	CIMMYT, Mexico	-	-	Elite line/germplasm	-
84.	EC576585	E3228	CIMMYT, Mexico	-	-	Elite line/germplasm	-
85.	EC190899	Cultivar No.37	CIMMYT, Mexico	-	-	Elite germplasm	-
86.	EC574849	9008	CIMMYT, Mexico	-	-	Elite line/germplasm	-
87.	EC576175	E10906	CIMMYT, Mexico	-	-	Elite line/germplasm	-
88.	IC582706	HPW240	Malan, Kangra (HP)	2009	-	Germplasm collection (Gehun Kanak)	-
89.	IC393878	WH157	CCSHAU, Hisar	1978	NP876/S308//CNO /8156	For saline/alkaline soil	Irrigated, timely sown conditions of NWPZ
90.	IC542544	PAU4088	PAU, Ludhiana	-	-	Germplasm collection	-
91.	IC566223	HS492	IARI RS, Shimla	2008	HPW42/CPAN2032/ UNATH K.S.	-	-
92.	IC342668	VKG21/72	Chatra, Jharkhand	2002	-	Germplasm collection	-
93.	IC535717	PBW139	PAU, Ludhiana	-	-	Germplasm collection	-
94.	IC553599	HI1544 (Purna)	IARI RS, Indore	2008	HINDI62/BOBWHI TE/CPAN 2099	Early maturing, field resistance to brown and black rusts	Irrigated, timely sown conditions of CZ
95.	EC277134	713	CIMMYT, Mexico	-	-	Elite line/germplasm	-
96.	NA	CUO/79/ Pru 11A	CIMMYT, Mexico	-	-	Elite line/germplasm	-

*NA-Not available; **BHU-**Banaras Hindu University, Varanasi, Uttar Pradesh; **CIMMYT-**International Maize and Wheat Improvement Center, Mexico; **CSUAT-**Chandra Shekhar Azad University of Agriculture and Technology, Kanpur, Uttar Pradesh; **CCSHAU-** CCS Haryana Agricultural University, Hisar, Haryana; **IARI-**Indian Agriculture Research Institute, New Delhi; **IARI RS:** IARI Regional station; **IIWBR-**Indian Institute of Wheat and Barley Research, Karnal, Haryana; **JAU**: Junagadh Agricultural University, Junagadh, Gujarat; **NDUAT-**Acharya Narendra Deva University of Agriculture and Technology, Ayodhya, Uttar Pradesh; **PAU-**Punjab Agricultural University, Ludhiana, Punjab; **PDKV-**Panjabaro Deshmukh Krishi Vidyapeeth, Akola, Maharashtra; **RARI-**Rajasthan Agricultural Research Institute, Jaipur, Rajasthan; **RARS**: Regional Agricultural Research Station, Vijapur, Gujarat; **USDA-**United States Department of Agriculture, USA; **VPKAS-**Vivekananda Parvatiya Krishi Anusandhan Sansthan, Almora, Uttarakhand; **HP**: Himachal Pradesh; **TN**: Tamil Nadu; **WB**: West Bengal.

Supplementary Table S2 │ ANOVA for combined ABD data from 18 morpho-physiological and yield contributing traits recorded in 96 accessions of bread wheat under non-stressed and heat-stressed environments during two crop seasons of years 2018-19 and 2019-20.

**Trait /**	**Non-stressed Environment**				**Heat-stressed Environment**			
**Source of variation ⟶**	**Year**	**Genotype**	**G × Y**	**Model**	**Year**	**Genotype**	**G × Y**	**Model**
Degree of freedom	1	95	95	191	1	95	95	191
Chlorophyll Content	6.89	39.67**	12.87	26.14**	0.53	44.72**	1.26	22.92**
CTD	191.49**	8.26	7.49	8.47	241.42**	4.44	3.21	5.18
NDVI	0.144**	0.003**	0.002*	0.003**	0.187**	0.008**	0.003**	0.007**
MSI (%)	585.30**	45.12	6.17	28.06	1016.06**	93.24**	5.97	54.17**
Plant Waxiness (0-10 scale)	8.53**	8.03**	0.59	4.35**	0.01	6.03**	0.06	3.03**
Leaf Rolling (0-10 scale)	0.72	3.57**	0.56	2.05**	0.03	3.56**	0.17	1.86**
Days to 50% Anthesis	320.25**	51.69**	6.44	30.34**	277.15**	35.72**	2.92	20.59**
GFD (days)	255.12**	13.46**	2.90	9.92**	484.91**	8.16**	2.26	8.02**
Plant Height (cm)	1252.37**	341.14**	32.67	193.41**	371.49**	309.45**	24.63**	168.12**
Peduncle Length (cm)	26.89	78.09**	7.25	42.68**	171.44**	60.86**	8.66**	36.06**
Flag Leaf Area (cm²)	749.86**	157.87**	44.25	104.00**	1736.81**	83.14**	22.88	61.59**
Spike Length (cm)	39.66**	2.87**	0.50	1.97**	1.51*	2.47**	0.67**	1.57**
Spikelets per Spike	2.56	4.52**	1.28	2.90**	0.56	4.63**	0.96	2.78**
Grain Length (mm)	0.72**	0.33**	0.03	0.18**	0.24**	0.32**	0.03**	0.18**
Grain Width (mm)	3.56**	0.08**	0.02	0.07**	3.31**	0.07**	0.02	0.06**
1000-Grain Weight (g)	138.41**	55.58**	11.32*	33.70**	1395.47**	46.04**	11.43	37.05**
Harvest Index (%)	1.91	53.49**	21.30	37.20	33.56	44.40**	20.05	32.36**
Grain Yield per Plot (g)	2600.86	36292.57**	563.68	18341.37**	6215.54	27016.67**	271.41	3598.04**

**, * significant at 0.01 and 0.05 probability level, respectively.

Supplementary Table S3 │ Combined adjusted mean values for 18 morpho-physiological and yield contributing traits recorded in 96 accessions of bread wheat over two years 2018-19 and 2019-20 under non-stressed environment.

Sl. No	Accession	CC	CTD (ºC)	NDVI	MSI (%)	PW	LR	DA (days)	GFP (days)	PH (cm)	PL (cm)	FLA (cm²)	SL (cm)	NSS	GL (mm)	GW (mm)	TGW (g)	HI (%)	GY (g/m²)
1.	Raj3765	22.4	8.6	0.635	56.3	5.8	5.2	91.6	35.1	99.9	38.1	42.1	11.7	19.0	7.24	3.52	43.0	42.2	552.5
2.	HD2932	25.4	8.8	0.625	69.3	7.9	6.9	90.8	35.5	98.3	35.6	30.6	11.2	19.8	6.59	3.69	41.7	44.8	614.7
3.	WR544	25.4	7.7	0.580	64.6	5.3	4.8	83.4	38.2	105.2	44.5	42.6	11.8	18.7	6.88	3.67	44.6	44.4	655.1
4.	HD2967	31.9	9.2	0.658	64.0	7.8	6.7	95.6	33.7	99.9	33.7	33.0	10.9	19.7	6.48	3.65	44.9	45.8	665.2
5.	EC574731	32.8	2.7	0.617	69.3	6.5	5.0	94.0	30.0	100.2	36.9	41.3	10.5	20.7	7.44	3.42	45.2	41.4	549.2
6.	EC576707	24.0	5.0	0.557	76.2	4.0	5.0	90.5	32.0	134.0	50.8	33.7	11.5	20.7	6.58	3.27	33.8	37.0	579.2
7.	IC252725	27.4	4.0	0.620	66.5	7.0	7.0	90.5	36.5	104.9	39.7	38.1	12.9	19.5	6.71	3.58	46.4	40.5	598.5
8.	IC252816	20.0	4.7	0.582	72.3	4.0	4.5	91.5	35.5	138.5	48.5	37.3	10.5	19.4	7.23	3.25	39.2	33.5	554.5
9.	IC277741	23.8	5.6	0.673	77.5	5.0	4.0	98.5	34.5	133.9	41.7	54.0	13.3	22.4	7.03	3.25	38.0	30.3	802.5
10.	IC536081	22.8	5.7	0.614	67.6	7.5	4.5	92.0	32.0	117.5	46.8	48.2	11.9	21.5	6.90	3.40	41.3	39.4	499.2
11.	IC279617	30.0	5.8	0.650	72.1	7.5	6.5	92.0	33.5	100.7	35.1	50.1	12.5	21.2	6.66	3.41	36.2	38.2	482.5
12.	IC535176	26.9	6.3	0.624	77.1	4.5	5.0	92.5	33.5	122.2	54.1	50.9	12.7	22.5	7.23	3.67	47.1	36.4	593.9
13.	IC401976	30.2	5.8	0.652	65.2	8.0	6.0	92.0	36.0	100.9	38.4	77.0	15.1	24.0	7.42	3.77	49.7	36.8	510.5
14.	IC539221	30.9	6.2	0.637	67.1	1.5	4.5	91.5	33.5	124.9	46.3	65.5	14.1	21.9	7.12	3.60	51.8	36.2	593.2
15.	IC539287	20.2	6.3	0.648	63.9	2.0	2.5	88.5	32.5	126.2	39.9	38.2	8.7	20.4	8.76	2.84	39.3	29.2	353.2
16.	IC539531	34.2	5.7	0.635	77.1	7.0	6.0	89.0	37.0	90.5	35.8	61.9	12.3	23.0	7.10	3.64	48.5	42.7	517.2
17.	IC443661	31.0	7.3	0.697	71.8	6.0	6.5	98.5	34.0	100.5	39.0	52.8	13.9	21.9	6.77	3.52	41.1	37.1	523.2
18.	EC534487	25.5	6.6	0.632	67.3	7.0	6.0	94.0	33.0	106.4	32.7	39.3	11.5	22.2	6.80	3.64	47.7	44.2	609.9
19.	IC416018	26.5	6.7	0.602	60.6	6.0	9.0	88.0	36.0	89.5	33.4	34.2	11.1	19.0	7.37	3.47	47.1	50.6	605.9
20.	IC416075	24.0	5.8	0.584	57.7	6.5	6.0	87.5	36.5	89.4	35.8	35.2	10.4	18.9	7.30	3.61	46.6	39.4	483.2
21.	IC416078	28.9	7.0	0.603	63.2	5.5	7.5	90.0	34.5	89.2	30.6	39.3	11.6	21.9	6.65	3.29	36.9	38.9	530.5
22.	IC416019	27.4	7.0	0.614	70.7	6.5	9.5	88.5	35.5	88.7	29.5	33.5	10.7	18.2	7.38	3.53	49.1	43.2	542.5
23.	IC446713	22.4	6.7	0.610	63.2	4.5	4.0	91.5	35.5	128.5	52.9	36.1	10.2	18.2	6.79	3.47	46.6	37.8	545.2
24.	IC075240	29.4	6.6	0.627	58.6	4.5	4.0	92.0	35.0	126.0	53.5	38.6	11.1	18.9	6.93	3.59	44.7	33.2	501.9
25.	EC178071	28.7	5.1	0.652	72.7	6.0	4.5	96.0	31.0	100.2	42.7	41.9	13.2	20.7	7.01	3.49	49.0	42.6	462.7
26.	IC542509	25.7	4.5	0.722	75.8	7.0	4.5	119.0	24.0	109.4	34.9	59.8	15.6	21.2	6.98	2.84	30.0	21.6	300.0

27.	IC252348	28.3	5.7	0.643	69.0	6.5	4.5	95.0	32.5	123.5	44.9	45.0	12.8	20.2	7.22	3.46	46.9	35.3	488.0
28.	IC543293	30.8	5.9	0.682	66.7	8.0	5.0	98.5	30.5	103.5	34.3	33.2	11.1	20.9	6.46	3.50	37.0	45.3	522.7
29.	IC128454	30.7	5.6	0.647	69.8	7.0	5.0	99.0	28.5	111.9	40.5	39.8	12.6	22.0	6.74	3.42	34.9	37.9	546.0
30.	IC416055	23.0	4.7	0.632	77.1	7.5	9.0	92.0	35.0	99.2	33.2	21.9	11.1	20.7	6.43	3.25	32.9	42.8	490.7
31.	IC111800	36.2	5.5	0.635	72.5	7.5	6.5	91.5	35.0	101.2	35.0	35.9	12.3	20.2	7.11	3.37	35.0	32.5	466.0
32.	IC111931	18.9	4.4	0.574	72.0	6.5	6.0	92.0	34.0	104.5	43.6	34.4	12.6	19.7	7.01	3.50	40.7	38.7	402.0
33.	EC576317	22.0	4.5	0.570	68.2	4.5	4.5	86.5	37.5	123.4	52.6	26.7	9.6	16.0	6.34	3.59	40.7	30.1	422.7
34.	EC577013	21.9	5.3	0.652	70.8	4.5	5.0	98.5	29.5	150.9	60.1	37.7	11.8	19.2	5.97	3.40	35.8	27.7	441.4
35.	EC414149	36.5	2.6	0.662	68.4	7.5	6.0	90.0	34.5	90.7	31.0	40.3	9.0	19.7	6.83	4.02	50.0	35.2	422.0
36.	IC252653	33.1	4.1	0.597	66.9	7.5	6.0	91.5	33.5	115.4	43.7	34.0	10.7	19.7	6.32	3.54	37.5	41.5	613.4
37.	IC252739	30.7	3.8	0.667	66.3	6.0	5.0	93.0	33.5	99.7	38.1	38.8	12.1	21.2	6.87	3.63	36.3	41.2	608.7
38.	IC335792	22.4	5.4	0.627	72.1	8.5	8.0	92.0	32.5	84.9	29.4	25.9	9.4	20.0	6.35	3.35	34.0	46.3	593.4
39.	IC543425	28.1	5.6	0.634	69.7	8.5	9.0	92.5	34.5	103.7	38.9	28.9	12.0	22.7	6.40	3.51	39.7	38.4	508.0
40.	IC402055	25.2	6.0	0.655	71.3	2.0	3.5	90.0	36.0	127.4	46.3	35.8	11.0	19.5	7.19	3.46	42.9	31.4	412.7
41.	IC265318	32.9	5.0	0.595	70.0	6.5	7.0	87.5	37.0	98.9	35.6	33.4	11.9	18.0	6.95	3.44	38.5	42.0	508.0
42.	IC445449	28.6	5.4	0.705	63.0	5.5	7.0	99.5	33.5	101.7	43.0	44.6	12.3	21.2	6.67	3.45	35.6	37.3	517.4
43.	IC528965	31.1	5.6	0.629	76.2	10.0	7.0	97.0	30.5	123.2	39.5	37.9	12.2	21.7	6.93	3.35	41.4	36.2	541.4
44.	IC549437	26.3	4.3	0.710	76.0	1.0	7.5	99.0	30.0	98.9	30.2	27.3	11.9	17.7	7.06	3.40	42.0	36.3	501.4
45.	IC144911	28.1	4.4	0.592	52.8	8.5	6.5	92.0	33.0	108.5	38.8	35.9	11.5	21.4	6.44	3.39	35.0	39.0	648.9
46.	IC542578	21.2	4.4	0.614	53.6	6.5	6.5	93.0	32.0	106.5	36.3	29.8	10.4	20.2	6.81	3.55	43.2	42.7	566.9
47.	IC535704	22.7	3.9	0.617	53.5	6.0	5.5	87.5	36.0	103.9	37.6	34.6	11.1	18.2	6.55	3.57	41.3	39.2	576.9
48.	EC542533	24.3	4.4	0.645	55.5	4.5	5.0	93.5	32.0	104.5	32.7	35.2	11.8	21.0	6.40	3.18	31.8	41.0	524.2
49.	IC542652	23.2	3.1	0.609	58.5	7.0	5.5	93.0	34.5	106.0	38.9	42.0	11.0	18.7	6.75	3.31	32.9	30.5	448.9
50.	IC536468	24.2	4.6	0.590	53.2	5.0	6.0	90.0	34.0	107.3	38.4	30.3	10.7	20.2	6.71	3.59	45.3	40.5	586.2
51.	IC536483	28.4	4.9	0.672	61.1	6.5	5.5	97.0	31.5	94.0	33.7	37.8	11.2	22.2	6.31	3.48	37.3	41.5	564.2
52.	EC574735	24.8	3.6	0.650	64.4	2.5	7.0	94.0	33.0	98.9	39.1	28.2	11.1	19.4	6.38	3.42	38.5	38.9	565.5
53.	IC531191	27.5	3.0	0.644	57.8	5.5	6.0	93.0	35.0	89.5	32.5	30.8	11.6	21.2	6.56	3.29	34.6	40.1	557.5
54.	IC333095	24.7	4.2	0.607	61.5	7.5	6.0	91.0	34.5	102.2	39.2	32.3	12.2	20.7	6.53	3.84	38.6	42.7	566.9
55.	IC572925	21.3	3.6	0.592	58.0	5.5	5.0	88.0	33.5	99.5	34.6	33.1	11.0	17.4	7.15	3.65	42.1	45.4	634.2

56.	IC252867	29.8	4.1	0.542	57.2	8.5	5.0	84.0	37.5	107.9	40.8	37.9	11.5	18.9	6.98	3.61	44.6	42.8	597.5
57.	IC524299	29.8	4.3	0.615	66.7	6.5	4.5	88.0	37.0	106.2	34.7	33.3	11.1	21.2	7.08	3.62	50.5	43.4	709.5
58.	IC573461	38.5	4.3	0.594	50.0	6.0	5.0	87.5	36.5	99.4	38.4	40.7	11.3	18.7	7.31	3.81	52.2	42.7	654.9
59.	IC252444	35.0	4.8	0.700	58.9	1.0	5.0	95.5	33.5	105.5	32.4	36.0	12.6	19.4	7.19	3.13	42.6	34.4	430.2
60.	IC529207	32.2	4.7	0.680	56.5	10.0	7.0	102.0	28.0	117.5	34.5	40.8	13.5	22.4	6.44	3.44	39.3	36.1	604.9
61.	IC290191	25.2	3.4	0.632	55.7	4.0	4.5	93.5	32.5	111.4	45.4	35.1	12.3	20.5	6.87	3.47	39.7	38.2	522.2
62.	IC112258	26.4	5.5	0.637	55.5	6.0	6.5	95.0	31.5	113.4	38.6	42.1	12.4	21.4	7.24	2.97	39.3	38.9	620.9
63.	IC627711	25.3	6.4	0.608	56.8	4.5	3.5	91.5	35.5	131.5	49.3	38.9	11.8	18.9	7.27	3.43	44.2	32.5	524.9
64.	IC443653	24.2	6.1	0.635	52.0	7.0	4.0	91.0	35.5	86.0	36.6	33.7	10.2	16.9	7.04	3.55	39.3	36.5	599.5
65.	IC252431	25.5	7.1	0.682	73.2	1.5	6.5	95.5	32.5	108.0	33.9	34.7	10.7	20.2	6.50	3.50	37.7	41.0	739.5
66.	IC252619	24.5	6.8	0.650	72.6	7.5	6.5	93.0	35.0	102.9	39.1	37.0	13.0	21.9	6.85	3.63	40.2	45.9	659.5
67.	IC529242	30.3	7.4	0.672	69.8	3.0	4.5	94.5	33.5	105.9	40.4	36.8	11.2	22.0	6.88	3.40	40.5	39.9	600.9
68.	IC536162	22.4	5.6	0.598	64.7	8.0	6.5	85.5	37.5	97.5	35.7	31.6	10.3	19.4	6.79	3.61	40.3	41.7	570.9
69.	IC536050	21.8	6.5	0.579	71.1	6.5	5.5	84.5	36.5	100.9	33.4	27.9	9.8	20.0	6.97	3.44	41.4	44.6	538.9
70.	IC252999	24.9	6.3	0.592	67.7	7.0	5.5	85.0	37.5	101.2	41.0	36.4	11.7	19.5	7.00	3.58	39.6	41.4	627.5
71.	IC443640	20.9	6.7	0.592	66.3	6.0	5.5	90.0	33.0	106.7	35.8	39.0	11.2	18.7	7.21	3.60	41.8	41.6	625.5
72.	IC445365	26.9	8.8	0.645	71.6	6.5	6.0	93.0	33.5	101.4	39.8	44.5	12.9	21.5	6.91	3.53	38.3	39.7	601.5
73.	IC303071	24.9	8.6	0.629	65.2	9.0	6.0	91.0	34.0	100.9	34.2	40.0	11.5	20.0	7.15	3.48	39.0	34.3	441.5
74.	IC252414	32.7	8.3	0.594	70.1	7.5	5.0	86.5	36.5	107.4	39.2	38.9	13.7	22.0	6.94	3.50	46.6	43.3	605.5
75.	IC372643	26.7	8.5	0.583	76.4	7.5	6.0	89.0	37.5	87.5	36.1	33.3	11.5	19.5	7.10	3.52	39.8	47.3	518.9
76.	IC252620	24.9	8.4	0.625	74.8	7.0	8.0	91.5	34.0	88.4	31.5	25.6	11.1	20.7	6.54	3.33	35.9	45.7	542.2
77.	IC240818	24.2	7.2	0.612	71.7	7.5	5.5	91.0	34.5	102.7	39.8	41.6	12.9	20.7	6.69	3.61	40.8	40.2	575.5
78.	IC401940	28.5	8.1	0.558	73.9	1.5	5.5	86.0	38.0	98.2	39.7	34.9	12.8	20.4	7.61	3.57	47.8	45.5	528.9
79.	IC443694	20.5	8.2	0.613	63.1	7.0	7.5	91.5	36.0	108.0	40.8	26.9	11.8	18.9	6.49	3.68	37.7	37.4	630.9
80.	IC542547	17.4	8.5	0.632	74.4	5.5	5.0	94.5	32.5	105.9	40.7	29.8	10.9	21.2	6.40	3.24	31.7	39.5	591.5
81.	EC190962	26.6	9.7	0.583	74.4	5.5	6.0	88.0	34.5	102.7	38.9	33.0	12.5	20.2	7.02	3.62	42.2	43.6	594.9
82.	EC576066	32.6	8.8	0.597	69.7	8.5	5.5	94.5	31.0	110.7	36.8	36.1	13.0	21.5	6.95	3.43	40.4	43.5	646.2
83.	EC573527	32.1	7.4	0.640	77.5	6.0	6.5	92.5	34.5	90.0	36.4	32.2	11.5	19.7	6.63	3.50	36.4	43.4	462.9
84.	EC576585	23.1	8.2	0.607	69.7	6.5	5.0	85.0	36.5	131.9	53.2	37.2	11.8	18.7	7.08	3.68	48.5	42.3	637.5

85.	EC190899	30.3	6.8	0.572	56.9	6.5	6.0	86.5	36.5	101.0	43.1	34.2	11.9	18.0	7.21	3.62	41.1	44.3	732.4
86.	EC574849	22.5	7.9	0.624	69.7	6.5	5.5	88.0	37.0	105.9	40.4	41.8	12.1	20.4	7.14	3.73	46.6	38.2	507.0
87.	EC576175	24.5	8.9	0.603	66.6	4.0	7.0	92.0	34.5	131.4	47.6	29.6	10.1	19.0	7.17	3.52	44.4	36.6	661.7
88.	IC582706	30.7	8.3	0.599	66.4	7.5	5.5	92.0	34.0	103.9	36.3	38.3	12.4	20.5	6.80	3.50	41.5	37.3	581.7
89.	IC393878	24.0	9.0	0.560	70.5	7.0	6.0	89.5	39.5	106.0	43.8	30.7	11.9	18.7	7.53	3.76	49.3	46.2	633.7
90.	IC542544	29.3	8.3	0.637	71.9	5.5	6.0	94.0	35.5	92.5	35.9	29.1	9.4	20.0	6.11	3.30	34.0	40.3	435.7
91.	IC566223	26.0	8.7	0.720	68.0	8.0	6.0	96.5	35.0	120.5	37.9	41.3	13.4	22.4	7.17	3.39	40.4	40.1	604.3
92.	IC342668	24.9	8.6	0.595	68.0	7.0	7.0	88.0	36.5	107.5	33.8	23.5	11.9	20.4	7.01	3.51	51.1	41.5	582.4
93.	IC535717	32.0	6.9	0.574	64.6	8.0	5.5	86.0	36.0	108.5	40.5	37.2	11.8	20.0	6.95	3.52	43.4	42.1	649.0
94.	IC553599	25.2	8.6	0.582	68.0	6.0	4.0	88.0	35.5	98.9	35.1	41.9	11.4	18.5	6.76	3.58	42.4	41.9	685.7
95.	EC277134	26.5	8.8	0.597	66.4	9.0	8.0	90.5	35.0	103.0	35.5	25.9	11.1	18.4	7.24	3.65	48.0	49.4	680.4
96.	CUO/79/ Pru11A	29.0	9.5	0.600	63.7	9.0	7.5	93.0	33.0	112.2	42.3	38.3	12.9	20.2	7.32	3.68	52.1	42.3	741.0

CC-Chlorophyll content, **CTD-**Canopy temperature depression, **NDVI-**Normalized difference vegetative index, **MSI-**Membrane stability index, **PW-**Plant waxiness, **LR-**Leaf rolling, **DA-**Days to 50 % anthesis, **GFP-**Grain filling Period, **PH-**Plant height, **PL-**Peduncle length, **FLA-**Flag leaf area, **SL-**Spike length, **NSS-**Number of spikelets per spike, **GL-**Grain length, **GW-**Grain width, **TGW-**Thousand grain weight, **HI-**Harvest index, **GY**-Grain yield.

Supplementary Table S4 │ Combined adjusted mean values for 18 morpho-physiological and yield contributing traits recorded in 96 accessions of bread wheat over two years 2018-19 and 2019-20 under heat-stressed environment.

Sl. No	Accession	CC	CTD (°C)	NDVI	MSI (%)	PW	LR	DA (days)	GFP (days)	PH (cm)	PL (cm)	FLA (cm^2)	SL (cm)	NSS	GL (mm)	GW (mm)	TGW (g)	HI (%)	GY (g/m^2)
1	Raj3765	21.2	7.1	0.470	63.5	6.6	5.4	74.4	28.6	91.9	33.4	30.4	10.9	18.8	7.15	3.34	38.7	36.7	472.0
2	HD2932	24.0	7.0	0.491	62.5	8.5	7.9	74.1	28.9	90.0	31.9	19.9	10.6	19.7	6.39	3.43	36.6	38.5	458.8
3	WR544	25.3	6.7	0.404	60.0	6.0	5.2	66.6	30.7	101.3	41.0	33.4	10.7	18.9	6.61	3.44	37.7	41.8	472.1
4	HD2967	34.0	8.0	0.558	66.3	8.0	6.4	76.8	28.8	90.8	31.3	25.1	10.0	19.8	6.30	3.43	36.2	35.7	518.7
5	EC574731	24.2	6.5	0.549	47.2	7.0	7.0	79.0	24.5	97.5	33.0	31.8	9.9	17.5	6.94	3.32	37.6	33.1	500.7
6	EC576707	20.3	8.0	0.452	48.0	7.0	5.0	73.5	29.0	118.2	44.9	21.6	10.8	18.9	6.38	3.18	32.4	28.9	534.7
7	IC252725	21.3	7.2	0.517	55.4	7.0	7.0	72.0	32.0	92.2	34.9	20.5	11.7	16.7	6.58	3.42	39.1	36.1	459.4
8	IC252816	15.5	7.8	0.492	61.4	6.0	4.0	76.0	29.0	124.9	41.3	21.2	10.1	17.7	7.13	3.24	36.4	29.8	431.4
9	IC277741	17.6	8.4	0.565	58.3	6.0	4.0	77.5	29.5	113.4	39.2	21.3	12.4	20.7	6.92	3.30	35.8	38.5	416.1
10	IC536081	22.1	8.6	0.458	53.0	8.0	6.0	75.5	26.5	103.7	39.6	24.3	11.4	18.9	6.62	3.30	35.1	34.5	316.1
11	IC279617	24.7	8.7	0.474	48.7	8.0	7.0	74.0	29.5	89.4	33.6	21.8	10.7	19.5	6.56	3.31	33.7	40.8	420.1
12	IC535176	23.3	9.0	0.509	50.4	6.0	6.0	73.5	30.0	112.2	53.8	29.7	11.5	19.5	6.95	3.55	45.0	39.0	532.1
13	IC401976	28.6	8.3	0.519	52.0	8.0	6.0	75.5	30.5	91.0	37.5	45.7	12.9	22.7	7.34	3.64	45.2	37.9	478.1
14	IC539221	26.1	10.2	0.538	45.7	2.0	5.0	74.0	29.0	121.4	43.9	32.5	13.5	20.5	6.89	3.43	46.6	35.3	468.7
15	IC539287	19.9	10.0	0.472	53.1	4.0	6.0	73.0	24.5	108.2	40.8	19.5	8.5	19.7	8.62	2.64	36.2	27.0	176.7
16	IC539531	26.0	9.4	0.473	59.3	8.0	6.0	70.5	31.5	85.4	35.4	27.0	11.0	20.9	6.76	3.48	42.6	39.7	452.1
17	IC443661	24.5	10.7	0.532	49.3	6.0	7.0	80.5	29.0	89.7	33.4	28.0	13.4	20.9	6.55	3.51	39.2	33.8	413.4
18	EC534487	23.2	10.9	0.494	58.7	8.0	7.0	76.5	29.0	94.0	31.1	20.1	11.1	20.7	6.65	3.32	37.6	37.6	496.1
19	IC416018	20.7	8.6	0.473	55.0	7.0	9.0	73.0	29.0	87.4	28.1	14.4	11.5	17.0	7.28	3.25	37.6	37.2	508.1
20	IC416075	21.9	6.2	0.382	50.3	7.0	6.0	69.5	31.0	80.0	31.9	25.6	8.6	17.9	7.11	3.43	41.9	33.8	458.1
21	IC416078	26.2	5.7	0.405	49.6	7.0	8.0	76.0	27.0	79.7	27.0	19.7	11.0	21.9	6.47	3.16	29.9	35.1	462.1
22	IC416019	17.2	7.2	0.440	59.6	7.0	10.0	74.5	27.5	84.7	28.3	17.1	9.4	16.9	7.08	3.30	36.8	39.9	490.1
23	IC446713	23.5	9.2	0.505	49.8	5.0	5.0	74.0	29.0	124.5	45.1	25.8	9.4	16.5	6.47	3.09	41.7	37.4	505.4
24	IC075240	23.9	9.0	0.569	50.3	6.0	5.0	75.5	28.0	125.4	46.5	25.9	9.3	17.4	6.82	3.19	39.9	33.8	460.1

25	EC178071	26.7	7.9	0.520	61.5	7.0	6.0	77.0	26.5	96.0	37.0	28.8	11.7	19.2	6.93	3.01	45.5	38.4	454.1
26	IC542509	26.0	12.0	0.642	67.5	8.0	5.0	94.0	24.0	103.7	31.8	51.7	12.0	18.5	6.73	2.88	26.2	23.4	287.4
27	IC252348	26.8	9.8	0.507	61.0	8.0	5.0	75.5	27.0	110.0	38.4	29.8	12.6	18.7	7.03	3.27	41.9	36.6	476.7
28	IC543293	22.2	9.3	0.524	56.8	8.0	5.0	81.0	26.0	88.4	29.0	21.7	10.4	20.5	6.12	3.28	31.8	25.2	341.4
29	IC128454	30.0	8.9	0.479	54.8	8.0	6.0	78.5	24.0	92.5	31.9	30.5	10.8	20.0	6.41	3.02	29.1	33.1	538.1
30	IC416055	18.8	8.3	0.483	60.6	9.0	10.0	74.5	28.0	92.7	32.5	14.3	10.0	18.0	6.16	3.13	29.0	38.4	418.7
31	IC111800	26.1	7.2	0.412	62.9	8.0	7.0	74.0	28.0	90.2	30.0	17.2	10.6	19.0	6.52	3.10	31.1	29.0	452.1
32	IC111931	17.2	7.1	0.390	59.2	7.0	6.0	75.5	27.5	89.7	35.8	28.9	10.9	18.5	6.60	3.27	31.6	28.8	347.4
33	EC576317	14.2	7.7	0.470	60.4	6.0	5.0	70.0	27.5	115.7	48.1	19.4	9.2	15.5	6.25	3.38	38.2	28.5	303.4
34	EC577013	18.9	7.7	0.545	62.8	6.0	5.0	82.0	23.5	125.0	46.1	24.5	9.8	17.7	5.85	3.22	28.9	25.0	242.1
35	EC414149	30.1	6.1	0.460	46.1	8.0	6.0	75.0	25.0	80.7	30.7	30.6	8.1	18.9	6.54	3.68	42.5	30.0	298.1
36	IC252653	24.0	6.3	0.482	55.9	8.0	7.0	74.0	26.5	99.2	38.1	18.0	11.0	17.7	6.23	3.18	28.5	38.3	481.4
37	IC252739	27.2	4.9	0.447	54.9	7.0	6.0	74.5	26.0	93.0	34.4	21.0	10.6	20.0	6.60	3.35	31.5	34.9	386.7
38	IC335792	19.8	6.3	0.479	59.7	8.0	8.0	73.5	28.0	80.7	29.9	14.2	9.3	18.0	6.17	3.03	29.9	38.9	539.4
39	IC543425	24.0	6.9	0.505	59.7	9.0	9.0	73.5	29.0	92.2	32.6	18.0	10.4	19.9	5.99	3.23	31.9	33.7	498.0
40	IC402055	21.5	6.4	0.542	55.0	3.0	4.0	73.0	29.5	116.5	35.5	23.2	10.5	18.5	7.06	3.37	40.9	35.5	328.7
41	IC265318	22.0	6.5	0.512	49.6	8.0	8.0	72.5	30.5	90.5	32.5	24.6	10.3	18.0	6.65	3.35	36.4	38.0	504.1
42	IC445449	20.6	6.9	0.600	55.0	6.0	9.0	83.0	25.0	91.7	32.5	24.3	11.8	20.2	6.37	3.10	29.0	28.2	343.4
43	IC528965	26.0	5.7	0.562	64.2	10.0	8.0	77.5	27.5	111.9	38.5	25.7	11.1	20.0	6.80	3.12	34.8	32.0	408.7
44	IC549437	20.2	6.6	0.632	48.5	2.0	7.0	79.5	27.0	85.0	27.5	15.7	11.5	17.0	7.07	3.27	36.3	29.0	333.4
45	IC144911	20.8	5.9	0.467	53.2	9.0	7.0	73.5	29.5	97.9	37.9	18.2	10.6	19.4	6.34	3.28	31.1	36.1	425.4
46	IC542578	17.0	6.1	0.480	61.2	7.0	7.0	75.0	26.5	95.7	32.8	18.6	10.2	17.9	6.72	3.27	35.5	37.0	474.1
47	IC535704	20.5	5.5	0.468	60.7	7.0	7.0	70.0	30.0	89.0	36.5	19.9	9.9	17.5	6.45	3.38	36.6	38.4	434.7
48	EC542533	20.9	5.5	0.488	64.3	5.0	6.0	76.0	27.0	95.3	32.9	25.0	11.1	18.2	6.26	3.14	28.3	33.6	419.4
49	IC542652	20.3	5.8	0.485	62.1	7.0	6.0	76.0	28.5	88.2	30.9	23.9	9.8	17.9	6.45	3.21	33.1	30.1	418.1
50	IC536468	23.8	5.3	0.474	62.1	6.0	7.0	73.5	29.0	100.8	36.4	21.4	9.9	19.5	6.46	3.48	38.0	42.0	435.4
51	IC536483	25.0	6.4	0.525	72.0	8.0	7.0	76.5	27.5	90.7	31.3	28.0	10.3	19.9	6.04	3.30	32.7	38.0	394.7
52	EC574735	19.2	6.4	0.555	69.9	4.0	8.0	77.0	28.5	93.5	36.6	21.4	9.7	16.5	6.29	3.23	31.0	33.5	458.7

53	IC531191	19.9	5.4	0.514	67.6	7.0	8.0	75.0	29.5	85.5	29.3	23.5	11.0	19.0	6.55	3.05	28.9	31.9	390.7
54	IC333095	24.9	6.1	0.529	66.8	8.0	7.0	74.0	27.5	90.9	35.5	20.1	11.1	20.0	6.40	3.58	33.4	45.0	430.1
55	IC572925	20.4	5.2	0.404	57.6	6.0	5.0	69.5	30.0	93.4	33.5	24.6	9.6	16.7	6.92	3.40	35.8	39.2	446.1
56	IC252867	24.2	4.7	0.407	43.7	8.0	7.0	69.0	29.0	104.7	37.1	22.9	10.8	17.7	6.83	3.28	38.6	39.8	438.7
57	IC524299	25.7	5.1	0.474	70.6	7.0	7.0	72.5	29.0	95.7	28.5	19.8	10.8	19.5	6.83	3.10	34.1	35.8	422.7
58	IC573461	27.8	4.8	0.480	59.2	6.0	6.0	75.0	29.5	94.2	31.3	21.9	10.3	16.7	6.97	3.23	31.1	30.2	462.7
59	IC252444	37.2	4.4	0.570	59.5	2.0	7.0	76.0	25.5	93.4	28.0	23.9	11.1	18.0	7.04	2.78	34.4	25.6	294.1
60	IC529207	32.1	6.2	0.549	63.2	10.0	8.0	80.5	26.5	99.2	30.2	31.7	12.1	21.0	6.40	3.32	35.7	33.6	527.4
61	IC290191	25.5	5.9	0.438	60.2	5.0	6.0	73.0	25.5	106.2	43.7	26.6	12.1	19.0	6.65	3.28	34.5	35.6	482.7
62	IC112258	20.9	7.2	0.464	61.0	7.0	7.0	78.5	24.5	102.4	32.2	24.5	11.9	20.4	6.91	2.75	30.0	30.2	424.7
63	IC627711	19.7	6.9	0.475	59.4	5.0	4.0	73.0	28.5	125.5	46.8	29.2	10.7	17.5	7.29	3.37	41.3	33.4	440.7
64	IC443653	20.7	6.5	0.410	60.2	7.0	5.0	69.5	31.0	73.2	30.7	23.8	9.4	16.5	6.72	3.47	33.3	48.0	417.4
65	IC252431	30.6	8.2	0.510	59.1	2.0	7.0	77.5	26.5	87.2	30.6	24.2	9.9	18.5	6.50	3.24	33.0	30.8	478.4
66	IC252619	22.8	6.8	0.445	66.2	7.0	7.0	75.0	26.5	85.0	34.3	24.5	12.5	21.5	6.41	3.36	32.2	34.9	441.7
67	IC529242	29.1	7.5	0.505	64.9	4.0	7.0	76.5	25.0	91.4	34.2	19.1	9.6	20.2	6.83	2.96	30.7	24.6	369.1
68	IC536162	21.5	6.0	0.409	64.5	8.0	6.0	68.5	26.5	85.5	29.7	23.4	8.9	16.5	6.72	3.34	37.2	32.8	376.4
69	IC536050	21.1	4.8	0.325	64.8	7.0	6.0	69.0	26.0	86.7	27.2	20.7	8.7	16.5	6.73	3.20	36.2	32.5	292.4
70	IC252999	18.4	4.7	0.315	54.2	8.0	7.0	68.5	26.0	93.4	38.5	26.8	11.1	18.7	6.74	3.33	34.4	33.4	443.7
71	IC443640	19.5	6.8	0.437	61.4	6.0	6.0	72.0	29.0	96.2	33.0	31.7	10.0	16.7	7.04	3.46	37.4	34.2	456.4
72	IC445365	24.9	6.5	0.488	65.5	6.0	7.0	75.5	28.0	88.5	36.1	22.3	10.9	19.7	6.77	3.36	34.4	36.0	400.4
73	IC303071	24.4	6.5	0.407	59.0	9.0	6.0	75.5	27.5	88.0	29.5	19.9	11.4	19.2	7.01	3.17	33.4	30.6	313.7
74	IC252414	27.1	5.8	0.499	61.1	7.0	5.0	73.0	28.0	100.2	33.5	23.8	12.6	21.4	6.57	3.23	37.4	33.5	403.1
75	IC372643	22.3	6.2	0.449	45.6	8.0	7.0	70.0	30.5	81.0	34.1	17.9	9.9	18.4	6.86	3.25	33.2	34.4	333.1
76	IC252620	22.8	7.3	0.510	63.2	7.0	8.0	74.5	28.0	78.2	27.8	17.9	11.0	19.5	6.50	3.19	28.9	31.8	347.7
77	IC240818	20.6	6.1	0.444	56.0	8.0	7.0	73.5	30.5	97.9	37.1	29.9	11.1	21.7	6.49	3.31	35.5	35.8	419.1
78	IC401940	19.3	6.5	0.489	56.1	3.0	6.0	70.5	31.5	91.5	33.4	28.1	12.1	17.2	7.14	3.28	43.0	39.2	391.7
79	IC443694	19.9	6.5	0.445	57.9	8.0	8.0	74.5	30.0	100.7	36.2	17.6	11.3	18.0	6.37	3.37	31.9	32.0	387.7
80	IC542547	13.5	6.7	0.572	65.1	6.0	6.0	77.5	27.5	93.5	32.6	15.2	10.4	19.7	6.05	3.00	26.1	35.3	391.1

81	EC190962	18.0	6.7	0.417	68.1	6.0	7.0	71.0	29.5	93.9	35.6	18.1	11.8	19.5	6.67	3.31	34.5	37.4	444.4
82	EC576066	26.1	6.5	0.483	64.5	9.0	6.0	79.0	25.5	101.7	36.0	27.8	9.9	18.5	6.71	3.21	34.8	27.7	376.4
83	EC573527	24.0	6.0	0.537	69.6	7.0	7.0	75.5	28.5	83.5	34.6	21.9	9.5	17.4	6.46	3.38	33.0	32.3	328.4
84	EC576585	19.8	6.6	0.365	54.3	7.0	5.0	68.0	28.5	127.0	48.1	31.3	10.9	17.9	6.76	3.52	44.7	37.6	353.7
85	EC190899	16.5	5.8	0.354	57.0	7.0	7.0	68.5	30.0	94.7	37.5	22.3	9.7	17.4	6.55	3.26	33.1	36.9	448.7
86	EC574849	15.2	6.5	0.490	69.0	7.0	5.0	71.5	30.0	95.0	36.3	27.1	10.6	20.2	6.81	3.45	41.4	34.8	353.4
87	EC576175	21.1	6.7	0.517	63.1	6.0	7.0	75.0	28.5	124.0	41.6	16.1	8.1	17.0	6.89	3.41	38.1	30.0	456.7
88	IC582706	19.3	6.2	0.455	66.5	8.0	7.0	75.5	28.0	92.9	34.1	26.5	11.3	19.4	6.63	3.42	34.2	34.5	459.4
89	IC393878	20.6	6.1	0.477	67.3	8.0	7.0	73.0	31.5	103.9	34.1	22.3	10.7	17.4	7.27	3.53	43.9	34.0	516.7
90	IC542544	21.7	5.2	0.489	72.6	7.0	6.0	75.5	29.0	77.5	31.1	18.1	9.1	19.9	6.11	2.90	24.9	35.8	331.4
91	IC566223	23.4	7.2	0.639	70.2	8.0	7.0	83.5	25.0	109.2	36.6	30.3	12.0	21.5	6.80	3.22	34.7	34.3	598.1
92	IC342668	21.1	5.8	0.469	69.1	8.0	7.0	72.5	30.0	98.9	30.4	19.4	10.2	18.9	6.43	3.24	40.7	33.2	450.7
93	IC535717	26.0	6.9	0.430	53.3	8.0	6.0	70.5	31.0	102.5	36.1	27.4	10.0	18.9	6.71	3.38	38.0	34.5	464.1
94	IC553599	19.7	6.4	0.465	63.3	6.0	4.0	72.0	29.5	92.2	33.2	24.6	9.9	16.4	6.68	3.30	39.3	38.7	424.7
95	EC277134	23.8	6.8	0.530	62.1	9.0	8.0	75.0	29.5	95.2	35.9	16.7	10.5	17.4	6.78	3.22	35.3	32.0	464.7
96	CUO/79/ Pru 11A	22.4	7.3	0.552	66.9	9.0	8.0	76.5	28.5	106.5	37.8	24.4	12.4	17.5	7.15	3.36	40.7	32.8	423.4

CC-Chlorophyll content, **CTD-**Canopy temperature depression, **NDVI-**Normalized difference vegetative index, **MSI-**Membrane stability index, **PW-**Plant waxiness, **LR-**Leaf rolling, **DA-**Days to 50 % anthesis, **GFP-**Grain filling Period, **PH-**Plant height, **PL-**Peduncle length, **FLA-**Flag leaf area, **SL-**Spike length, **NSS-**Number of spikelets per spike, **GL-**Grain length, **GW-**Grain width, **TGW-**Thousand grain weight, **HI-**Harvest index, **GY-** Grain yield.

Supplementary Table S5 | ANOVA for TGW and grain yield over four environments based on stability analysis.

**Sources of variation**	**D.F.**	**TGW (MSS)**	**Grain Yield (MSS)**
Genotypes (G)	95	89.54**	19206.70**
Environment (E)	3	1589.07**	614125.02**
Genotype × Environment (G × E)	285	11.57	2796.45**
Environment (E) + (G × E)	288	28.00**	9164.46**
Environment (Linear)	1	4767.22**	1842375.06**
G × E (Linear)	95	9.53	18197.54**
Pooled deviation	192	12.46	148.88
Total	383	43.27	11655.34

** significant at 0.01 level of probability; **TGW**: 1000-grain weight, **MSS**: Mean sum of squares.

Supplementary Table S6 │ Mean performance and stability parameters of 96 bread wheat accessions for 1000-grain weight and grain yield based on Eberhart and Russell (1966) model.

Sl. No.	Accessions	Thousand grains weight (g)			Grain yield (g/m^2)		
		μ (mean)	β_i	S^2D_i	μ (mean)	β_i	S^2D_i
1.	RAJ3765	40.8	0.80	4.03	514.2	0.61	35.67
2.	HD2932	39.2	1.02	7.19	538.1	1.14	49.90
3.	WR544	41.1	0.99	3.10	562.8	1.31	25.24
4.	HD2967	40.5	1.25	3.38	592.7	1.03	46.12
5.	EC574731	42.0	0.99	0.22	520.7	0.43	62.51
6.	EC576707	33.7	0.15	2.20	552.1	0.36	37.58
7.	IC252725	43.3	0.97	3.18	525.2	1.07	27.60
8.	IC252816	38.3	0.56	7.28	490.4	0.95	22.91
9.	IC277741	37.5	0.68	42.16	597.6	2.74	76.59
10.	IC536081	38.8	0.46	17.02	401.9	1.36	7.88
11.	IC279617	35.6	0.29	8.29	448.7	0.53	56.35
12.	IC535176	46.7	0.19	0.94	556.0	0.47	61.91
13.	IC401976	48.0	0.73	4.67	493.3	0.28	21.89
14.	IC539221	49.8	0.86	3.33	529.0	0.94	12.34
15.	IC539287	38.4	-0.07	17.53	261.5	1.25	15.98
16.	IC539531	46.1	0.75	2.11	478.3	0.50	49.48
17.	IC443661	40.8	-0.06	1.18	463.9	0.85	24.38
18.	EC534487	43.2	1.24	2.62	551.6	0.92	80.85
19.	IC416018	43.0	0.89	13.29	553.8	0.78	36.99
20	IC416075	44.9	0.84	6.03	465.8	0.28	114.68
21.	IC416078	34.0	1.31	8.70	493.0	0.57	44.85
22.	IC416019	43.6	1.71	1.29	510.0	0.41	55.05
23.	IC446713	44.7	0.62	1.02	522.7	0.32	11.41
24.	IC075240	42.9	0.88	6.98	474.5	0.33	63.50
25.	EC178071	48.8	0.95	13.11	444.9	-0.23	247.57
26.	IC542509	29.7	1.02	36.45	274.1	-0.29	455.28
27.	IC252348	46.0	1.38	16.61	480.1	-0.15	529.35
28.	IC543293	36.0	1.38	15.66	424.0	1.14	353.21
29.	IC128454	33.6	1.05	1.91	543.6	-0.22	376.52
30.	IC416055	32.6	0.96	7.30	445.4	0.29	677.95
31.	IC111800	34.7	1.15	24.15	457.9	-0.02	131.26
32	IC111931	37.7	1.66	1.05	369.9	0.22	321.60
33.	EC576317	41.0	0.37	0.37	360.3	0.62	567.00
34.	EC577013	33.9	1.39	3.24	335.1	1.24	425.31
35.	EC414149	47.9	1.45	13.85	352.7	0.69	479.75
36.	IC252653	34.6	1.42	8.61	541.4	0.77	384.38
37.	IC252739	35.5	1.05	4.62	491.3	1.41	423.58
38.	IC335792	33.6	1.02	7.37	559.7	0.20	446.45
39.	IC543425	37.4	1.32	0.09	487.2	-0.56	319.85
40.	IC402055	43.5	0.21	1.34	365.1	0.43	369.43
41.	IC265318	39.1	0.71	11.30	500.7	-0.15	363.40
42.	IC445449	33.9	0.79	4.79	425.9	1.09	291.42
43.	IC528965	39.7	1.39	9.27	471.8	0.81	237.82
44.	IC549437	40.7	1.23	24.94	411.3	1.03	388.82
45.	IC144911	33.3	0.81	0.05	537.0	1.56	79.27
46.	IC542578	39.5	1.63	2.68	518.5	0.65	8.90
47.	IC535704	39.1	0.69	6.14	503.9	1.00	13.17
48.	EC542533	30.3	0.64	1.22	468.2	0.71	34.06

49.	IC542652	33.2	0.51	19.49	431.5	0.20	10.09
50.	IC536468	41.9	1.19	2.76	507.7	1.05	24.20
51.	IC536483	35.3	1.14	12.55	476.0	1.18	30.91
52.	EC574735	35.0	1.09	7.17	510.2	0.75	9.67
53.	IC531191	32.0	0.75	8.37	472.7	1.19	17.58
54.	IC333095	36.2	1.02	6.23	494.8	0.94	32.03
55.	IC572925	39.2	0.88	20.80	541.1	1.37	55.73
56.	IC252867	41.9	0.88	11.79	516.1	1.12	16.25
57.	IC524299	42.5	1.56	117.81	565.8	2.02	114.97
58.	IC573461	41.8	2.86	46.15	558.5	1.39	34.86
59.	IC252444	38.7	2.06	14.30	363.7	1.00	46.11
60.	IC529207	37.7	1.06	26.18	563.5	0.55	5.10
61.	IC290191	37.3	1.12	1.93	500.5	0.26	9.76
62.	IC112258	34.9	1.33	12.70	522.5	1.41	35.37
63.	IC627711	43.0	0.88	6.78	482.5	0.61	5.94
64.	IC443653	36.5	1.31	33.10	508.1	1.31	30.44
65.	IC252431	34.1	0.76	1.61	607.9	2.05	269.05
66.	IC252619	35.0	1.39	1.74	545.9	1.70	112.16
67.	IC529242	34.3	1.56	2.87	482.2	1.82	169.29
68.	IC536162	37.5	0.82	12.49	471.0	1.55	170.89
69.	IC536050	37.6	0.84	1.94	414.6	1.90	144.98
70.	IC252999	35.7	0.90	4.28	534.1	1.49	224.54
71.	IC443640	38.4	0.87	4.85	538.2	1.36	167.26
72.	IC445365	35.1	1.12	20.33	499.5	1.61	227.49
73.	IC303071	34.9	1.53	32.85	374.9	1.02	69.33
74.	IC252414	40.7	1.45	4.76	501.7	1.56	73.93
75.	IC372643	35.3	1.18	12.68	423.2	1.48	166.22
76.	IC252620	31.1	1.01	0.37	442.3	1.55	166.20
77.	IC240818	36.9	0.90	2.11	495.4	1.29	209.65
78.	IC401940	44.1	0.57	5.15	455.9	1.11	113.08
79.	IC443694	33.6	0.89	5.34	505.4	1.93	240.81
80.	IC542547	27.6	1.02	4.22	486.9	1.57	104.72
81.	EC190962	37.1	1.16	5.65	515.3	1.21	114.00
82.	EC576066	36.4	0.33	11.99	509.6	2.10	230.58
83.	EC573527	33.5	0.50	1.65	393.7	1.12	201.03
84.	EC576585	45.4	0.75	7.26	492.9	2.19	178.97
85.	EC190899	35.8	1.81	3.98	588.3	2.18	155.19
86.	EC574849	42.8	1.04	3.30	432.2	1.25	230.28
87.	EC576175	40.0	1.32	3.41	557.9	1.62	176.60
88.	IC582706	36.5	1.01	19.14	517.5	1.00	109.69
89.	IC393878	45.3	1.21	3.85	572.2	0.96	109.62
90.	IC542544	28.2	0.85	61.15	380.9	0.86	100.43
91.	IC566223	36.3	0.76	23.13	589.5	0.04	119.09
92.	IC342668	44.6	1.31	34.64	515.2	1.04	72.22
93.	IC535717	39.4	0.69	17.82	553.6	1.45	104.97
94.	IC553599	39.6	0.66	2.56	550.5	1.98	57.60
95.	EC277134	40.4	1.32	71.39	570.8	1.69	154.00
96.	CUO/79/Pru 11A	45.1	1.10	75.79	580.5	2.37	102.43
	**Pop Mean**	**38.6**	-	-	**489.4**	-	-

Supplementary Table S7│Heat-stress tolerant and susceptible bread wheat accessions identified based on grain yield along with important physiological and yield contributing traits.

Sl. No.	Accession	CC		CTD (°C)		NDVI		MSI (%)		PW (0-10 scale)		LR (0-10 scale)		GFP (days)		FLA (cm^2)		Plant Height (cm)		PL (cm)		SL (cm)		TGW (g)		Grain Yield (g/m^2)	
		NS	HS	NS	HS	NS	HS	NS	HS	NS	HS	NS	HS	NS	HS	NS	HS	NS	HS	NS	HS	NS	HS	NS	HS	NS	HS
**(a)**	**Highly tolerant**																										
1.	IC566223	26.0	23.4	8.7	7.2	0.720	0.639	68.0	70.2	8.0	8.0	6.0	7.0	35.0	25.0	41.3	30.3	120.5	109.2	37.9	36.6	13.4	12.0	40.4	34.7	604.3	598.1
2.	IC335792	22.4	19.8	5.4	6.3	0.627	0.479	72.1	59.7	8.5	8.0	8.0	8.0	32.5	28.0	25.9	14.2	84.9	80.7	29.4	29.9	9.4	9.3	34.0	29.9	593.4	539.4
3.	EC576707	24.0	20.3	5.0	8.0	0.557	0.452	76.2	48.0	4.0	7.0	5.0	5.0	32.0	29.0	33.7	21.6	134.0	118.2	50.8	44.9	11.5	10.8	33.8	32.4	579.2	534.7
4.	IC535176	26.9	23.3	6.3	9.0	0.624	0.509	77.1	50.4	4.5	6.0	5.0	6.0	33.5	30.0	50.9	29.7	122.2	112.2	54.1	53.8	12.7	11.5	47.1	45.0	593.9	532.1
5.	IC529207	32.2	32.1	4.7	6.2	0.680	0.549	56.5	63.2	10.0	10.0	7.0	8.0	28.0	26.5	40.8	31.7	117.5	99.2	34.5	30.2	13.5	12.1	39.3	35.7	604.9	527.4
6.	IC128454	30.7	30.0	5.6	8.9	0.647	0.479	69.8	54.8	7.0	8.0	5.0	6.0	28.5	24.0	39.8	30.5	111.9	92.5	40.5	31.9	12.6	10.8	34.9	29.1	546.0	538.1
7.	IC393878	24.0	20.6	9.0	6.1	0.560	0.477	70.5	67.3	7.0	8.0	6.0	7.0	39.5	31.5	30.7	22.3	106.0	103.9	43.8	34.1	11.9	10.7	49.3	43.9	633.7	516.7
8.	IC416018	26.5	20.7	6.7	8.6	0.602	0.473	60.6	55.0	6.0	7.0	9.0	9.0	36.0	29.0	34.2	14.4	89.5	87.4	33.4	28.1	11.1	11.5	47.1	37.6	605.9	508.1
9.	IC446713	22.4	23.5	6.7	9.2	0.610	0.505	63.2	49.8	4.5	5.0	4.0	5.0	35.5	29.0	36.1	25.8	128.5	124.5	52.9	45.1	10.2	9.4	46.6	41.7	545.2	505.4
10.	IC265318	32.9	22.0	5.0	6.5	0.595	0.512	70.0	49.6	6.5	8.0	7.0	8.0	37.0	30.5	33.4	24.6	98.9	90.5	35.6	32.5	11.9	10.3	38.5	36.4	508.0	504.1
11.	EC574731	32.8	24.2	2.7	6.5	0.617	0.549	69.3	47.2	6.5	7.0	5.0	7.0	30.0	24.5	41.3	31.8	100.2	97.5	36.9	33.0	10.5	9.9	45.2	37.6	549.2	500.7
**(b)**	**Tolerant**																										
12.	IC543425	28.1	24.0	5.6	6.9	0.634	0.505	69.7	59.7	8.5	9.0	9.0	9	34.5	29.0	28.9	18.0	103.7	92.2	38.9	32.6	12.0	10.4	39.7	31.9	508.0	498.0
13.	EC534487	25.5	23.2	6.6	10.9	0.632	0.494	67.3	58.7	7.0	8.0	6.0	7.0	33.0	29.0	39.3	20.1	106.4	94.0	32.7	31.1	11.5	11.1	47.7	37.6	609.9	496.1
14.	IC416019	27.4	17.2	7.0	7.2	0.614	0.440	70.7	59.6	6.5	7.0	9.5	10.0	35.5	27.5	33.5	17.1	88.7	84.7	29.5	28.3	10.7	9.4	49.1	36.8	542.5	490.1
15.	IC290191	25.2	25.5	3.4	5.9	0.632	0.438	55.7	60.2	4.0	5.0	4.5	6.0	32.5	25.5	35.1	26.6	111.4	106.2	45.4	43.7	12.3	12.1	39.7	34.5	522.2	482.7
16.	IC252653	33.1	24.0	4.1	6.3	0.597	0.482	66.9	55.9	7.5	8.0	6.0	7.0	33.5	26.5	34.0	18.0	115.4	99.2	43.7	38.1	10.7	11.0	37.5	28.5	613.4	481.4
17.	IC401976	30.2	28.6	5.8	8.3	0.652	0.519	65.2	52.0	8.0	8.0	6.0	6.0	36.0	30.5	77.0	45.7	100.9	91.0	38.4	37.5	15.1	12.9	49.7	45.2	510.5	478.1
18.	IC252348	28.3	26.8	5.7	9.8	0.643	0.507	69.0	61.0	6.5	8.0	4.5	5.0	32.5	27.0	45.0	29.8	123.5	110.0	44.9	38.4	12.8	12.6	46.9	41.9	488.0	476.7
19.	IC542578	21.2	17.0	4.4	6.1	0.614	0.480	53.6	61.2	6.5	7.0	6.5	7.0	32.0	26.5	29.8	18.6	106.5	95.7	36.3	32.8	10.4	10.2	43.2	35.5	566.9	474.1
20.	IC539221	30.9	26.1	6.2	10.2	0.637	0.538	67.1	45.7	1.5	2.0	4.5	5.0	33.5	29.0	65.5	32.5	124.9	121.4	46.3	43.9	14.1	13.5	51.8	46.6	593.2	468.7
21.	IC075240	29.4	23.9	6.6	9.0	0.627	0.569	58.6	50.3	4.5	6.0	4.0	5.0	35.0	28.0	38.6	25.9	126.0	125.4	53.5	46.5	11.1	9.3	44.7	39.9	501.9	460.1
22.	IC539531	34.2	26.0	5.7	9.4	0.635	0.473	77.1	59.3	7.0	8.0	6.0	6.0	37.0	31.5	61.9	27.0	90.5	85.4	35.8	35.4	12.3	11.0	48.5	42.6	517.2	452.1
23.	IC443661	31.0	24.5	7.3	10.7	0.697	0.532	71.8	49.3	6.0	6.0	6.5	7.0	34.0	29.0	52.8	28.0	100.5	89.7	39.0	33.4	13.9	13.4	41.1	39.2	523.2	413.4
**(c)**	**Susceptible**																										
24.	IC443640	20.9	19.5	6.7	6.8	0.592	0.437	66.3	61.4	6.0	6.0	5.5	6.0	33.0	29.0	39.0	31.7	106.7	96.2	35.8	33.0	11.2	10.0	41.8	37.4	625.5	456.4
25.	IC535717	32.0	26.0	6.9	6.9	0.574	0.430	64.6	53.3	8.0	8.0	5.5	6.0	36.0	31.0	37.2	27.4	108.5	102.5	40.5	36.1	11.8	10.0	43.4	38.0	649.0	464.1

26.	IC573461	38.5	27.8	4.3	4.8	0.594	0.480	50.0	59.2	6.0	6.0	5.0	6.0	36.5	29.5	40.7	21.9	99.4	94.2	38.4	31.3	11.3	10.3	52.2	31.1	654.9	462.7
27.	IC252999	24.9	18.4	6.3	4.7	0.592	0.315	67.7	54.2	7.0	8.0	5.5	7.0	37.5	26.0	36.4	26.8	101.2	93.4	41.0	38.5	11.7	11.1	39.6	34.4	627.5	443.7
28.	IC572925	21.3	20.4	3.6	5.2	0.592	0.404	58.0	57.6	5.5	6.0	5.0	5.0	33.5	30.0	33.1	24.6	99.5	93.4	34.6	33.5	11.0	9.6	42.1	35.8	634.2	446.1
29.	EC576175	24.5	21.1	8.9	6.7	0.603	0.517	66.6	63.1	4.0	6.0	7.0	7.0	34.5	28.5	29.6	16.1	131.4	124.0	47.6	41.6	10.1	8.1	44.4	38.1	661.7	456.7
30.	EC277134	26.5	23.8	8.8	6.8	0.597	0.530	66.4	62.1	9.0	9.0	8.0	8.0	35.0	29.5	25.9	16.7	103.0	95.2	35.5	35.9	11.1	10.5	48.0	35.3	680.4	464.7
31.	IC252619	24.5	22.8	6.8	6.8	0.650	0.445	72.6	66.2	7.5	7.0	6.5	7.0	35.0	26.5	37.0	24.5	102.9	85.0	39.1	34.3	13.0	12.5	40.2	32.2	659.5	441.7
32.	IC252414	32.7	27.1	8.3	5.8	0.594	0.499	70.1	61.1	7.5	7.0	5.0	5.0	36.5	28.0	38.9	23.8	107.4	100.2	39.2	33.5	13.7	12.6	46.6	37.4	605.5	403.1
33.	IC144911	28.1	20.8	4.4	5.9	0.592	0.467	52.8	53.2	8.5	9.0	6.5	7.0	33.0	29.5	35.9	18.2	108.5	97.9	38.8	37.9	11.5	10.6	35.0	31.1	648.9	425.4
34.	IC252431	25.5	30.6	7.1	8.2	0.682	0.510	73.2	59.1	1.5	2.0	6.5	7.0	32.5	26.5	34.7	24.2	108.0	87.2	33.9	30.6	10.7	9.9	37.7	33.0	739.5	478.4
**(d)**	**Highly Susceptible**																										
35.	EC190899	30.3	16.5	6.8	5.8	0.572	0.354	56.9	57.0	6.5	7.0	6.0	7.0	36.5	30.0	34.2	22.3	101.0	94.7	43.1	37.5	11.9	9.7	41.1	33.1	732.4	448.7
36.	IC443694	20.5	19.9	8.2	6.5	0.613	0.445	63.1	57.9	7.0	8.0	7.5	8.0	36.0	30.0	26.9	17.6	108.0	100.7	40.8	36.2	11.8	11.3	37.7	31.9	630.9	387.7
37.	IC529242	30.3	29.1	7.4	7.5	0.672	0.505	69.8	64.9	3.0	4.0	4.5	7.0	33.5	25.0	36.8	19.1	105.9	91.4	40.4	34.2	11.2	9.6	40.5	30.7	600.9	369.1
38.	IC553599	25.2	19.7	8.6	6.4	0.582	0.465	68.0	63.3	6.0	6.0	4.0	4.0	35.5	29.5	41.9	24.6	98.9	92.2	35.1	33.2	11.4	9.9	42.4	39.3	685.7	424.7
39.	IC524299	29.8	25.7	4.3	5.1	0.615	0.474	66.7	70.6	6.5	7.0	4.5	7.0	37.0	29.0	33.3	19.8	106.2	95.7	34.7	28.5	11.1	10.8	50.5	34.1	709.5	422.7
40.	EC576066	32.6	26.1	8.8	6.5	0.597	0.483	69.7	64.5	8.5	9.0	5.5	6.0	31.0	25.5	36.1	27.8	110.7	101.7	36.8	36.0	13.0	9.9	40.4	34.8	646.2	376.4
41.	CUO/79/ Pru11A	29.0	22.4	9.5	7.3	0.600	0.552	63.7	66.9	9.0	9.0	7.5	8.0	33.0	28.5	38.3	24.4	112.2	106.5	42.3	37.8	12.9	12.4	52.1	40.7	741.0	423.4
42.	EC576585	23.1	19.8	8.2	6.6	0.607	0.365	69.7	54.3	6.5	7.0	5.0	5.0	36.5	28.5	37.2	31.3	131.9	127.0	53.2	48.1	11.8	10.9	48.5	44.7	637.5	353.7
43.	IC536050	21.8	21.1	6.5	4.8	0.579	0.325	71.1	64.8	6.5	7.0	5.5	6.0	36.5	26.0	27.9	20.7	100.9	86.7	33.4	27.2	9.8	8.7	41.4	36.2	538.9	292.4
44.	IC277741	23.8	17.6	5.6	8.4	0.673	0.565	77.5	58.3	5.0	6.0	4.0	4.0	34.5	29.5	54.0	21.3	133.9	113.4	41.7	39.2	13.3	12.4	38.0	35.8	802.5	416.1
**(e)**	**National check varieties**																										
45.	Raj3765 (C1)	22.4	21.2	8.6	7.1	0.635	0.470	56.3	63.5	5.8	6.6	5.2	5.4	35.1	28.6	42.1	30.4	99.9	91.9	38.1	33.4	11.7	10.9	43.0	38.7	552.5	472.0
46.	HD2932 (C2)	25.4	24.0	8.8	7.0	0.625	0.491	69.3	62.5	7.9	8.5	6.9	7.9	35.5	28.9	30.6	19.9	98.3	90.0	35.6	31.9	11.2	10.6	41.7	36.6	614.7	458.8
47.	WR544 (C3)	25.4	25.3	7.7	6.7	0.580	0.404	64.6	60.0	5.3	6.0	4.8	5.2	38.2	30.7	42.6	33.4	105.2	101.3	44.5	41.0	11.8	10.7	44.6	37.7	655.1	472.1
48.	HD2967 (C4)	31.9	34.0	9.2	8.0	0.658	0.558	64.0	66.3	7.8	8.0	6.7	6.4	33.7	28.8	33.0	25.1	99.9	90.8	33.7	31.3	10.9	10.0	44.9	36.2	665.2	518.7
	Minimum	17.4	13.5	2.6	4.4	0.542	0.315	50.0	43.7	1.0	2.0	2.5	4.0	24.0	23.5	21.9	14.2	84.9	73.2	29.4	27.0	8.7	8.1	30.0	24.9	300.0	176.7
	Maximum	38.5	37.2	9.7	12.0	0.722	0.642	77.5	72.6	10.0	10.0	9.5	10.0	39.5	32.0	77.0	51.7	150.9	127.0	60.1	53.8	15.6	13.5	52.2	46.6	802.5	598.1
	Pop mean	26.8	22.7	6.2	6.9	0.624	0.484	66.6	59.4	6.2	6.9	5.8	6.5	34.2	28.1	37.4	23.8	106.6	96.9	39.2	35.1	11.7	10.6	41.5	35.5	562.2	423.6
	LSD (5%)	2.5	2.3	1.7	1.5	0.033	0.032	5.0	4.7	0.8	0.8	0.6	0.7	2.0	1.9	4.2	3.1	4.9	4.8	2.9	2.2	0.6	0.5	2.1	2.6	49.0	21.0

NS: Non stressed environment; **HS:** Heat stressed environment; **LSD:** Least significant difference at 0.05 level of probability; **CC-**Chlorophyll content, **CTD-**Canopy temperature depression, **NDVI-**Normalized difference vegetation index, **MSI-**Membrane stability index**, PW-**Plant waxiness, **LR-**Leaf rolling, **GFP-**Grain filling period, **FLA-**Flag leaf area, **PL:** Peduncle length; **SL-**Spike length, **TGW-**1000-grains weight..

Supplementary Table S8 │Promising accessions identified for different traits for both non-stressed (NS) and heat-stressed (HS) environments.

Traits	Environ-ments	Top 10 promising accessions for different morpho-physiological and yield-related traits
Chlorophyll Content (CCI)	NS	IC573461, EC414149, IC111800, IC252444, IC539531, IC252653, IC265318, EC574731, IC252414, EC576066 (**>32.5**)
	HS	IC252444, HD2967, IC529207, IC252431, EC414149, IC128454, IC529242, IC401976, IC252739, IC252414 (**>27.0**)
CTD (°C)	NS	EC190962, CUO/79/Pru11A, HD2967, IC393878, EC576175, HD2932, IC445365, EC576066, EC277134, IC566223 (**>8.5 °C**)
	HS	IC542509, EC534487, IC443661, IC539221, IC539287, IC252348, IC539531, IC543293, IC446713, IC535176 (**>9.0 °C**)
NDVI	NS	IC542509, IC566223, IC549437, IC445449, IC252444, IC443661, IC543293, IC252431, IC529207, IC536483 (**>0.6720**)
	HS	IC542509, IC566223, IC549437, IC445449, IC542547, IC252444, IC075240, IC277741, IC528965, HD2967 (**>0.560**)
MSI (%)	NS	IC252816, EC573527, IC535176, IC539531, IC416055, IC372643, EC576707, IC528965, IC549437, IC542509 (**>76.0%**)
	HS	IC542544, IC536483, IC524299, IC566223, EC574735, EC573527, IC342668, EC574849, IC393878, CUO/79/ Pru 11A (**>67.0%**)
Plant Waxiness (0-10 scale)	NS	IC528965, IC529207, IC303071, EC277134, CUO/79/Pru11A, IC335792, IC543425, IC144911, IC252867, EC576066 (**>8.5**)
	HS	IC528965, IC529207, CUO/79/Pru 11A, EC277134, IC416055, IC543425, IC144911, IC303071, EC576066, HD2932 (**>8.5**)
Leaf Rolling (0-10 scale)	NS	IC416019, IC416018, IC416055, IC543425, IC335792, IC252620, EC277134, IC416078, IC549437, IC443694 (**>7.5**)
	HS	IC416055, IC416019, IC416018, IC543425, IC445449, IC265318, IC528965, IC443694, EC277134, CUO/79/ Pru 11A (**>8.0**)
Days to 50% Anthesis	NS	WR544, IC252867, IC536050, IC252999, EC576585, IC536162, IC401940, IC535717, EC576317, IC252414 (**< 86.0 days**)
	HS	WR544, EC576585, IC536162, IC252999, EC190899, IC252867, IC536050, IC416075, IC572925, IC443653 (**<70.0 days**)
Grain Filling Period (days)	NS	IC393878, WR544, IC401940, EC576317, IC252867, IC536162, IC252999, IC372643, IC539531, IC524299 (**>37.0 days**)
	HS	IC252725, IC539531, IC401940, IC393878, IC416075, IC443653, IC535717, WR544, IC401976, IC265318 (**>30.5 days**)
Plant Height (cm)	NS	EC577013, IC252816, EC576707, IC277741, EC576585, IC627711, EC576175, IC446713, IC402055, IC539287 (**>126.0 cm**)
	HS	EC576585, IC627711, IC075240, EC577013, IC252816, IC446713, EC576175, IC539221, EC576707, IC402055 (**>116.0**)
Peduncle Length (cm)	NS	EC577013, IC535176, IC075240, EC576585, IC446713, EC576317, EC576707, IC627711, IC252816, EC576175 (**>47.0 cm**)
	HS	IC535176, EC576317, EC576585, IC627711, IC075240, EC577013, IC446713, EC576707, IC539221, IC290191 (**>44.0 cm**)
Flag Leaf Area (cm^2)	NS	IC401976, IC539221, IC542509, IC539531, IC277741, IC443661, IC535176, IC279617, IC536081, IC252348 (**>45.0**)
	HS	IC542509, IC401976, WR544, IC539221, EC574731, IC529207, IC443640,

		EC576585, EC414149, IC128454 (**>30.0 cm^2**)
Spike Length (cm)	NS	IC542509, IC401976, IC539221, IC443661, IC252414, IC529207, IC566223, IC277741, EC178071, EC576066 (**>13.0 cm**)
	HS	IC539221, IC443661, IC401976, IC252348, IC252414, IC252619, CUO/79/ Pru 11A, IC277741, IC529207, IC401940 (**> 12.0 cm**)
NSS	NS	IC401976, IC539531, IC543425, IC535176, IC277741, IC529207, IC566223, EC534487, IC128454, IC252414 (**> 22.0**)
	HS	IC401976, IC416078, IC240818, IC252619, IC566223, IC252414, IC529207, IC539531, IC443661, IC539221 (**> 20.0**)
Grain Length (mm)	NS	IC539287, IC401940, IC393878, EC574731, IC401976, IC416018, IC416019, IC573461, IC627711, CUO/79/ Pru 11A (**> 7.0 mm**)
	HS	IC539287, IC401976, IC627711, IC416018, IC393878, Raj3765, CUO/79/Pru11A, IC401940, IC252816, IC416075 (**> 7.0 mm**)
Grain Width (mm)	NS	EC414149, IC333095, IC573461, IC401976, IC393878, EC574849, HD2932, IC443694, EC576585, CUO/79/ Pru 11A (**>3.7 mm**)
	HS	EC414149, IC401976, IC333095, IC535176, IC393878, EC576585, IC443661, IC539531, IC536468, IC443653 (**>3.5 mm**)
1000-Grain Weight (g)	NS	IC573461, CUO/79/ Pru 11A, IC539221, IC342668, IC524299, EC414149, IC401976, IC393878, IC416019, EC178071 (**>49.0 g**)
	HS	IC539221, EC178071, IC401976, IC535176, EC576585, IC393878, IC401940, IC539531, EC414149, IC416075 (**> 42.0 g**)
Harvest Index (%)	NS	IC416018, EC277134, IC372643, IC335792, IC393878, IC252619, HD2967, IC252620, IC401940, IC572925 (**>45.0 %**)
	HS	IC443653, IC333095, IC536468, WR544, IC279617, IC416019, IC252867, IC539531, IC572925, IC401940 (**> 39.0 %**)
Grain Yield (g/m^2)	NS	IC277741,CUO/79/Pru11A, IC252431, EC190899, IC524299, IC553599, EC277134, HD2967, EC576175, IC573461 (**> 655 g**)
	HS	IC566223, IC128454, IC335792, EC576707, IC535176, IC529207, IC543425, HD2967, IC393878, IC416018 (**> 508 g**)
